# Two‐Photon Endomicroscopy for Image‐Guided Magnetic Micro‐Agents

**DOI:** 10.1002/smll.75051

**Published:** 2026-08-11

**Authors:** Juan J. Huaroto, Gert‐willem Römer, Ivo M. Vellekoop, Sarthak Misra

**Affiliations:** ^1^ Surgical Robotics Laboratory Department of Biomechanical Engineering University of Twente Enschede The Netherlands; ^2^ Chair of Laser Processing Department of Mechanics of Solids Surfaces & Systems (MS3) University of Twente Enschede The Netherlands; ^3^ Biomedical Photonic Imaging Group Faculty of Science and Technology University of Twente Enschede The Netherlands

**Keywords:** fluorescence imaging, magnetic actuation, microrobotics, two‐photon endomicroscopy

## Abstract

Magnetic microrobotics holds promise for minimally invasive biomedical applications, yet imaging in this field still relies predominantly on external modalities. Endoscopic imaging offers an alternative route for in situ visualization and micro‐agent guidance, but its use in microrobotics has so far remained limited to standard endoscopic modalities. This study demonstrates two‐photon endomicroscopy (TPE) for real‐time fluorescence imaging of magnetically actuated micro‐agents. Dynamic experiments under rotating magnetic fields support motion analysis across multiple actuation frequencies. In addition, dual‐color imaging enables analysis of mixed magnetic and non‐magnetic micro‐agent populations labeled with distinct fluorescent dyes. Imaging through biological tissue shows that magnetic micro‐agents remain detectable through a 140 μm rat mammary gland tissue layer. The feasibility of image‐guided re‐centering of a HeLa cell spheroid within the TPE field of view is also shown, compensating for displacement induced by the magnetic micro‐agents. These results extend TPE from passive observation to active image‐guided functionality in microrobotic systems.

## Graphical abstract

Image‐guided manipulation of magnetic micro‐agents requires localized fluorescence feedback capable of resolving dynamic interactions in confined environments. This study introduces a two‐photon endomicroscopy platform for real‐time motion tracking, multicolor micro‐agent discrimination, and imaging through biological tissue. Evaluation of magnetic micro‐agent interactions with a HeLa cell spheroid demonstrates image‐guided lateral re‐centering, connecting high‐resolution fluorescence visualization with magnetic actuation.

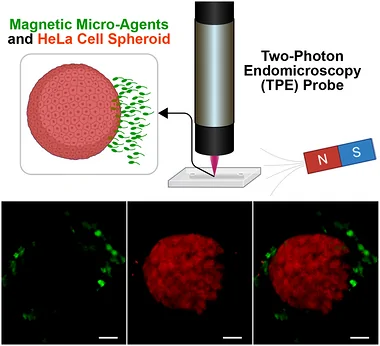

## 1 Introduction

The need for precise interventions within confined anatomical regions has driven the development of microrobotics for minimally invasive diagnosis and therapy [1, 2, 3, 4]. Engineered micro‐agents have been explored as carriers for drugs, cells, and genes in applications ranging from targeted chemotherapy to reproductive medicine [5, 6, 7, 8]. However, their successful therapeutic deployment requires reliable navigation through complex physiological environments, such as the digestive, circulatory, and reproductive systems [9]. Magnetic [10], acoustic [11], and optical fields [12] provide powerful strategies for remote micro‐agent actuation. Yet, in vivo controlled guidance depends on imaging modalities capable of providing real‐time and informative feedback during actuation [13].

Among actuation strategies, magnetism is particularly attractive because time‐varying magnetic fields and gradients enable diverse steering and locomotion modes [14, 15]. In many minimally invasive scenarios, magnetically actuated micro‐agents are intended to operate near a target site for tasks such as localized therapeutic delivery and interaction with biological samples [16, 17, 18, 19]. In this setting, in situ visualization becomes a valuable complementary modality for monitoring micro‐agent position, proximity to biological structures, and their interactions during actuation.

From the imaging perspective, most reported approaches in microrobotics rely on external systems based on established modalities such as x‐ray computed tomography [20], positron emission tomography [21], fluoroscopy [22], single‐photon emission computed tomography [23], ultrasound (US) [24], photoacoustic imaging (PAI) [25], magnetic resonance imaging [26], optical coherence tomography (OCT) [27], and magnetic particle imaging [28]. While these techniques enable non‐invasive visualization, they typically involve trade‐offs among spatial resolution, temporal resolution, and penetration depth that limit their effectiveness for precise micro‐agent tracking and guidance [13].

Endoscopic imaging offers an alternative route by moving the imaging modality closer to the site of interest and enabling in situ visualization [16, 29, 30]. Endoscopic modalities span a wide range of spatial‐resolution and imaging‐depth trade‐offs. Fluorescence endomicroscopy and OCT provide high spatial resolution at shallow imaging depths, whereas endoscopic US and PAI enable deeper imaging with comparatively lower spatial resolution. These capabilities support clinical applications such as tissue inspection, cancer detection, mucosal screening, and deep‐tissue imaging [31, 32, 33, 34, 35]. These systems are primarily optimized for tissue assessment, where motion is often intrinsic, slowly varying, or manageable through averaging, motion correction, and post‐processing. Microrobotics introduces a different operating regime, in which imaging targets are micrometer‐scale, move under external actuation, and require real‐time feedback for guidance [4]. Despite the rapid development of clinical endoscopic imaging, microrobotic applications have so far relied on standard endoscopes [16]. In this context, fluorescence endomicroscopy is particularly attractive because fluorescent labeling can provide contrast for distinguishing micro‐agents from surrounding biological structures. However, fluorescence‐based endomicroscopy remains underdeveloped for tracking and guiding magnetically actuated micro‐agents.

Two‐photon endomicroscopy (TPE) extends fluorescence endoscopy by exploiting nonlinear absorption to achieve intrinsically localized excitation, thereby limiting out‐of‐focus signals and reducing background fluorescence in scattering tissue [36, 37, 38]. Fiber‐based TPE probes have advanced rapidly in recent years for applications such as in vivo tissue imaging and neural activity imaging, establishing their potential as compact, high‐resolution imaging tools for minimally invasive use [39, 40, 41]. In parallel, two‐photon microscopy has enabled in vivo visualization during acoustic navigation and continuous imaging of magnetic micro‐agents through biological tissue [42, 43]. These developments position two‐photon imaging as a promising modality for microrobotics, while leaving open the use of TPE for in situ imaging and guidance of magnetically‐actuated micro‐agents. In this setting, effective image guidance requires dual‐color, real‐time imaging to distinguish micro‐agents from biological structures and resolve their interactions during actuation. Furthermore, because these interactions unfold within a limited imaging field, active repositioning is also important for maintaining micro‐agents and biological structures in view throughout actuation.

To address these requirements, this study demonstrates two‐photon endomicroscopy (TPE) for real‐time fluorescence imaging of magnetically actuated micro‐agents. The presented approach supports dual‐color visualization and motion analysis of differently labeled micro‐agents, imaging through biological tissue, and image‐guided re‐centering of a biological structure. Figure 1A depicts the proposed TPE system concept for visualizing magnetic micro‐agents and other fluorescently labeled structures during actuation. Figure 1B shows the corresponding experimental implementation, in which a fiber‐based TPE probe provides distal imaging, and a permanent magnet drives actuation. The magnetic micro‐agents used here are discrete fragments produced by sonication of Coumarin 6‐labeled electrospun precursor fibers containing ${\mathrm{Fe}}_{3}O_{4}$ nanoparticles (Figure 1C). Representative dual‐color TPE images of magnetic micro‐agents interacting with a HeLa cell spheroid, used here as a 3D cellular model, are shown in Figure 1D.

**FIGURE 1:**
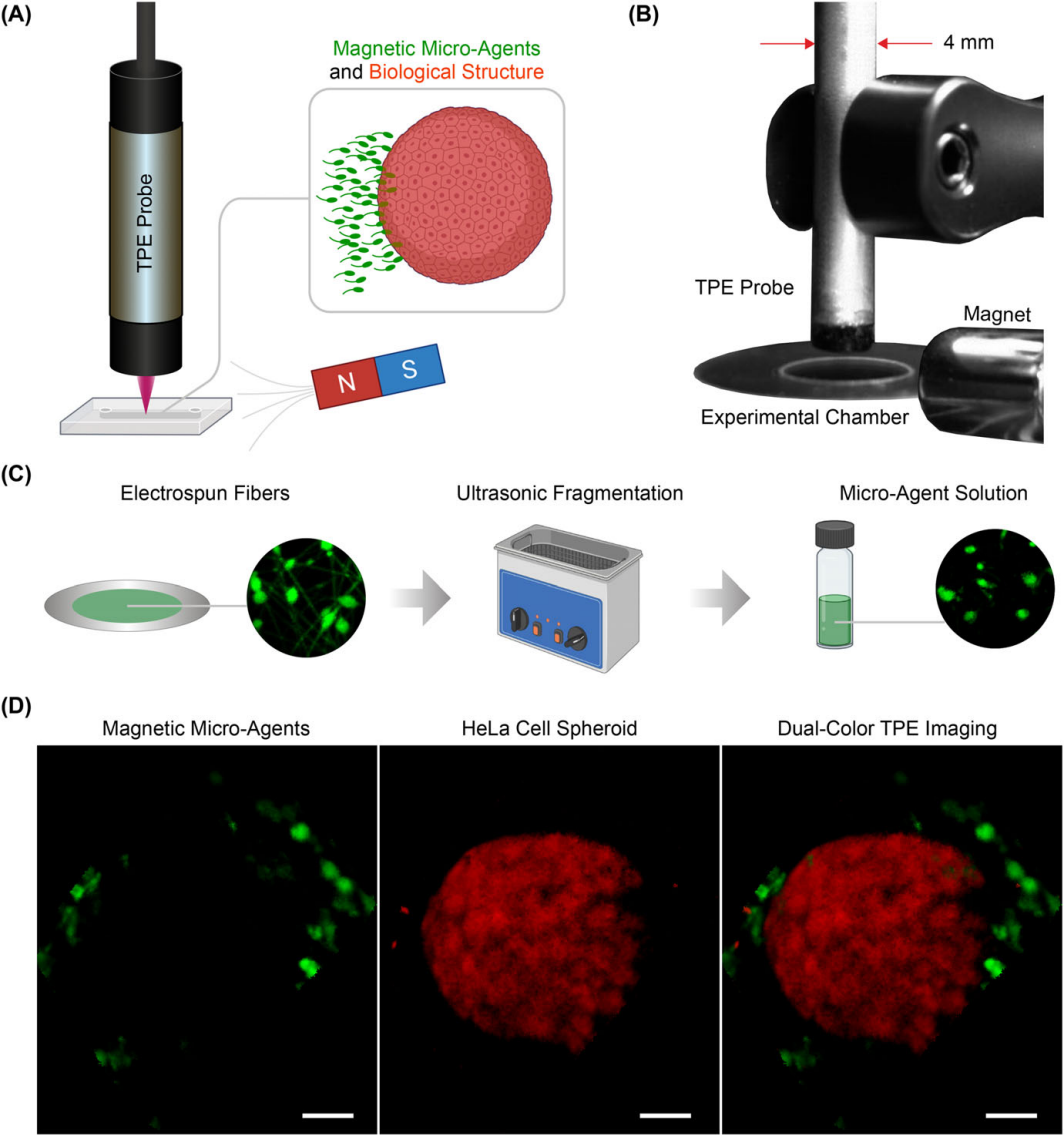
Two‐photon endomicroscopy (TPE) system for imaging of magnetic micro‐agents and biological structures. (A) Conceptual schematic of the experimental approach. A fiber‐based TPE probe enables fluorescence imaging of magnetic micro‐agents (green) and a fluorescently labeled biological structure (red), while external magnetic fields remotely actuate the micro‐agents. (B) Experimental implementation showing the distal TPE probe positioned adjacent to a permanent magnet used to generate magnetic fields for micro‐agent actuation. (C) Fabrication workflow of fluorescent magnetic micro‐agents. Electrospun precursor fibers are fragmented by ultrasonication to generate an aqueous suspension of micro‐agents. (D) Representative two‐photon endomicroscopy images showing magnetic micro‐agents (green), a HeLa cell spheroid used here as a 3D cellular model (red), and the merged dual‐color TPE image. Scale bars = 50 μm.

## 2 Results and Discussion

This section presents the TPE system and the main experimental results used to assess its performance for real‐time fluorescence imaging of magnetic micro‐agents. Section 2.1 provides a concise overview of the TPE system and its key operating characteristics, while Section 2.2 characterizes the fluorescence and morphology of the electrospun precursor fibers, including the effects of ${\mathrm{Fe}}_{3}O_{4}$ incorporation. Sections 2.3– 2.5 demonstrate TPE for motion analysis under magnetic actuation, imaging through biological tissue, and image‐guided re‐centering of a biological structure. Further details of the TPE probe, magnetic micro‐agents, and experimental procedures are provided in Section 4. .

### 2.1 Endomicroscopy System

The TPE system enables real‐time fluorescence imaging and supports simultaneous dual‐color detection through two spectral channels (Figure 2A). A femtosecond pulsed laser centered at 900 nm is delivered through a double‐clad fiber to the distal probe, and the generated fluorescence is collected through the same optical pathway and separated into two spectral channels for dual‐color imaging.

**FIGURE 2:**
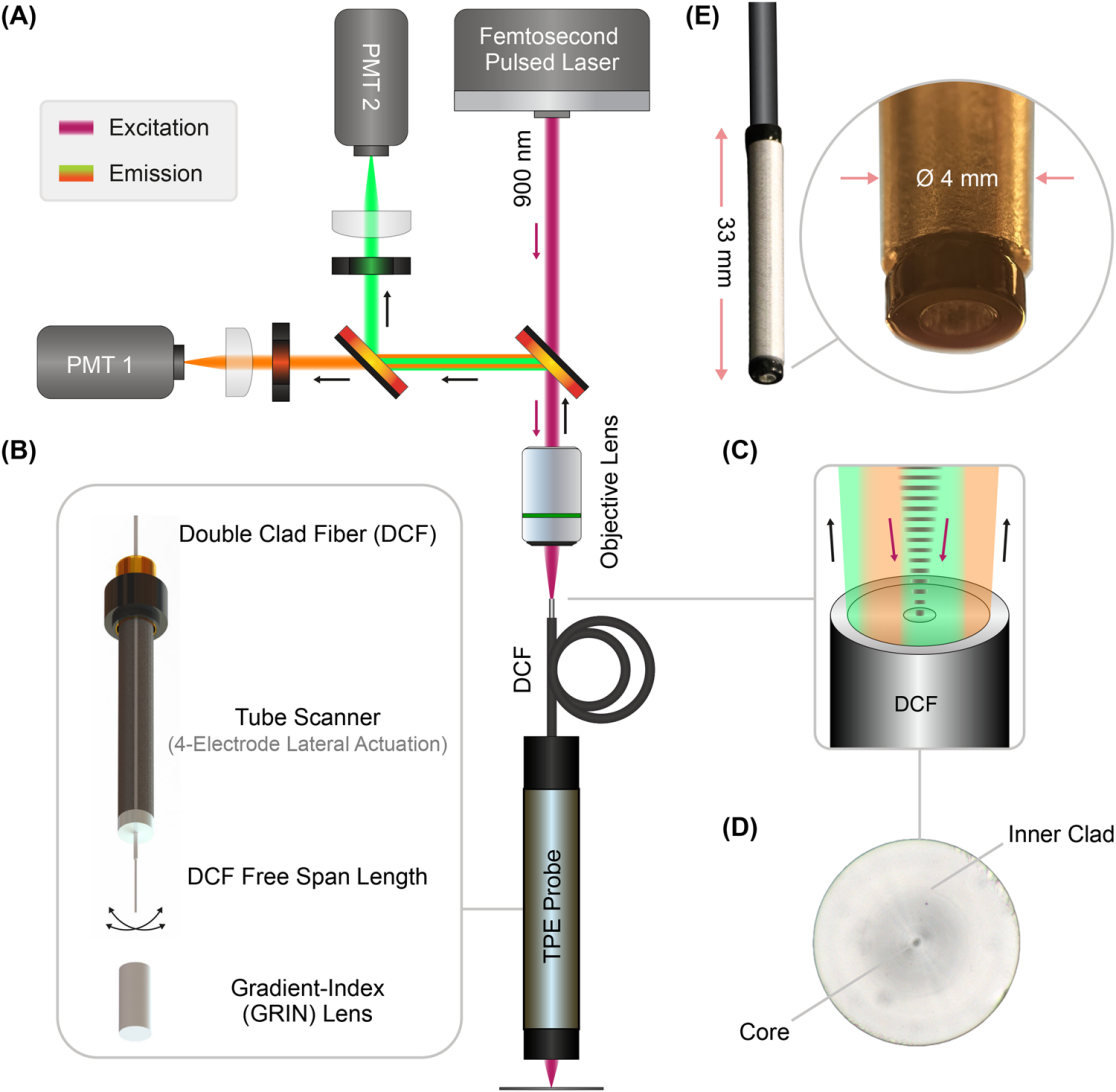
Optical configuration and probe architecture of the two‐photon endomicroscopy (TPE) system. (A) Optical layout of the TPE system. Femtosecond laser excitation at 900 nm is coupled into a double‐clad fiber (DCF) through the objective lens and delivered to the distal probe. Fluorescence generated at the sample is collected through the same fiber and separated into two spectral detection channels using dichroic optics and photomultiplier tubes (PMT1 and PMT2) for dual‐color imaging. (B) Distal probe architecture showing the four‐electrode piezoelectric tube scanner, free‐span DCF length, and gradient‐index (GRIN) lens. Two‐dimensional lateral scanning is achieved by oscillatory actuation of the assembled DCF–tube scanner, while the GRIN lens focuses the excitation beam into the sample. (C) Schematic of the double‐clad fiber operating principle. Excitation light propagates through the fiber core, while fluorescence emission is collected through the inner clad. (D) Microscopy image of the double‐clad fiber cross‐section showing the core and inner clad. (E) Photograph of the fabricated TPE probe, showing a distal tip diameter of approximately 4 mm and a rigid length of 33 mm.

The distal probe combines a piezoelectric tube scanner, a double‐clad fiber (DCF), and a GRIN lens (Figure 2B). Lateral scanning is produced by oscillatory actuation of the assembled DCF–tube scanner, while the GRIN lens focuses the excitation beam into the sample and collects the emitted fluorescence. The DCF geometry and operating principle are illustrated in Figure 2C,D, and a photograph of the fabricated distal probe is shown in Figure 2E.

Figure 3A summarizes the distal optical configuration of the DCF–GRIN lens assembly, including the DCF tip–GRIN lens gap ($l_{0}$), the effective numerical aperture, and the focused beam diameter. Physical‐optics simulations are used to guide the selection of $l_{0}$ for efficient two‐photon excitation. While the full simulation analysis spans $l_{0}$ = 1.0–4.5 mm (Figure S2), the final probe design is selected within $l_{0}$ = 2.5–3.0 mm range to balance centered focusing performance with off‐axis beam propagation during scanning. The selected configuration is excited by a laser source centered at 900 nm with a measured bandwidth of 9 nm (Figure 3B). Under these conditions, the probe delivers a focused distal spot with a lateral full width at half maximum of approximately 2.1 μm (Figure 3C), and beam propagation measurements support an effective numerical aperture of approximately 0.26 in air (Figure 3D).

**FIGURE 3:**
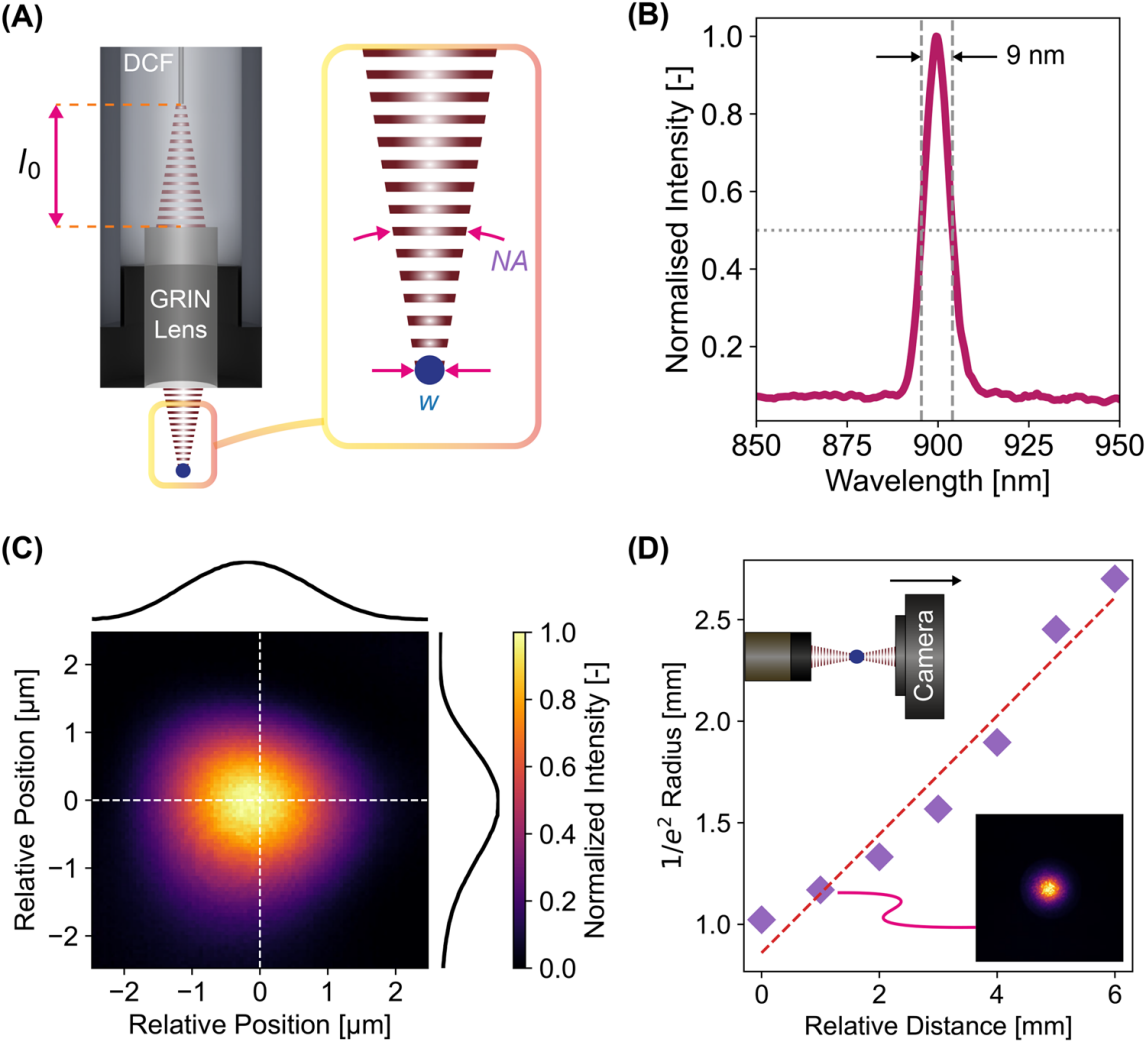
Optical characterization of the two‐photon endomicroscopy probe. (A) Schematic of probe geometry showing the the fiber‐to‐lens gap ($l_{0}$), focal spot size (w) and effective numerical aperture (NA). (B) Normalized laser emission spectrum centered at 900 nm, showing a 9 nm spectral bandwidth. (C) Lateral intensity profile at the focal plane used to estimate the probe point‐spread function. The measured focal spot has a full width at half maximum of approximately 2.1 μm. (D) Beam propagation measurements showing the evolution of the $1/e^{2}$ beam radius with axial distance from the probe tip. The dashed line indicates a linear fit used to estimate beam divergence. The inset shows a representative beam profile at one axial position. The fitted slope corresponds to an effective numerical aperture of approximately 0.26 in air.

The scanning characteristics of the assembled probe are then characterized experimentally to identify the operating conditions and usable scan extent (Figure S3). The responses along the two lateral axes show resonance peaks near 810 Hz, consistent with the finite‐element analysis of a DCF free length of 11.5 mm (Figure S4). Operation near this resonance yields relatively large scan amplitudes at drive voltages below the tube scanner limit of 200 V. For stable operation, the orthogonal electrodes are driven at 825 and 820 Hz with corresponding peak‐to‐peak voltages of 147.4 and 122.5 V, generating a Lissajous scan with robust spatial coverage across the scanned region. Under these conditions, the scan achieves lateral extents of 232 and 316 μm along the two axes at a 5 Hz image rate.

### 2.2 Engineering and Characterization of Electrospun Precursor Fibers

Having established the optical and scanning performance of the TPE probe, the system is next used to characterize electrospun precursor fibers that later serve as the source material for magnetic micro‐agents generated by ultrasonic fragmentation. The fibers are electrospun from polystyrene (PS) solutions in dimethylformamide (DMF) containing fluorescent dyes and magnetic nanoparticles, while the polymer concentration is varied to produce either straight or beaded morphologies (Figure 4A; detailed fabrication parameters are provided in Section 4. Coumarin 6 (C6) and Nile Red (NR) are selected as fluorescent dyes because both generate detectable two‐photon fluorescence under 900 nm excitation and provide partially separated spectral signatures for dual‐channel imaging.

**FIGURE 4:**
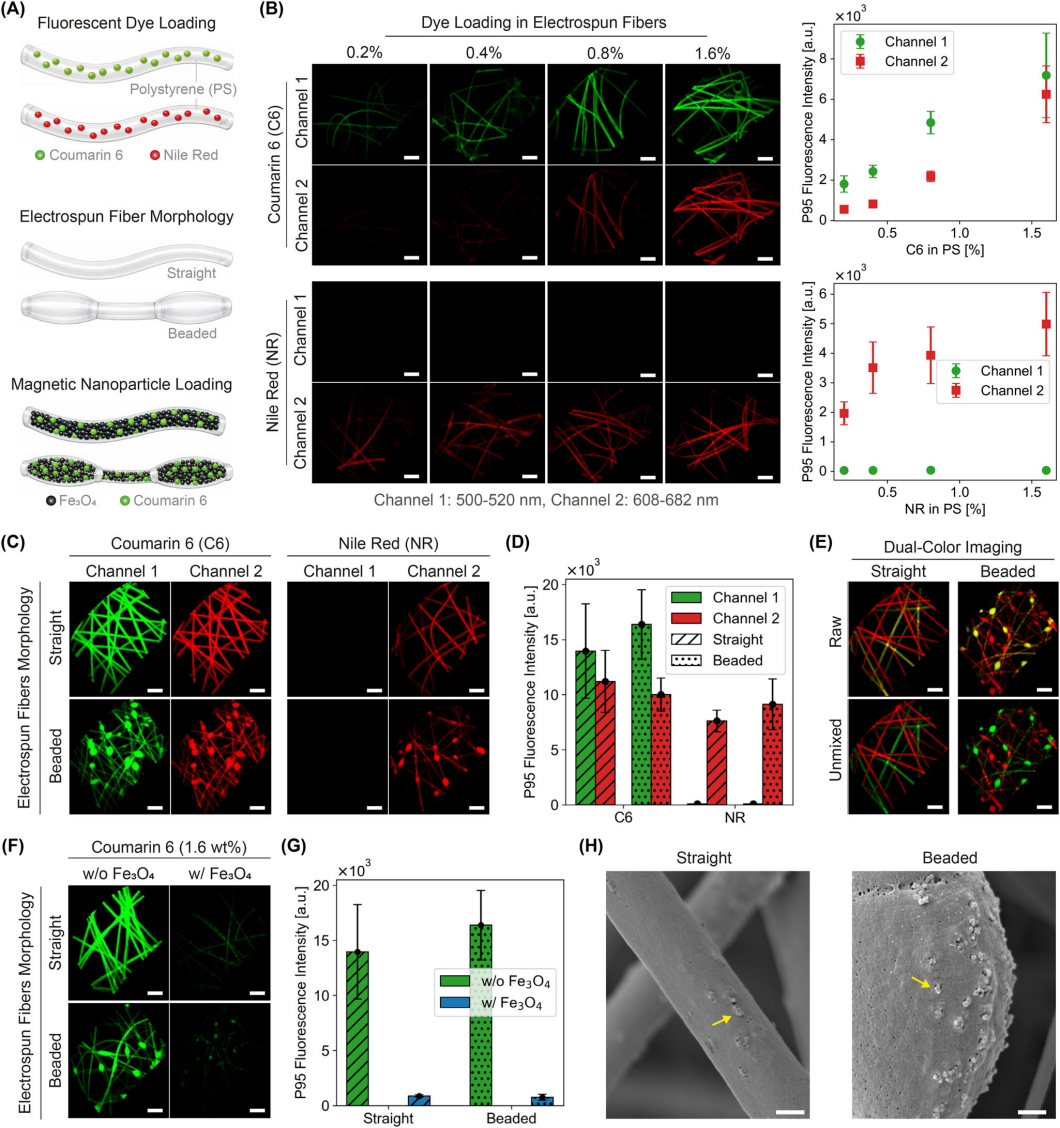
Engineering and optical characterization of electrospun precursor fibers used to generate magnetic micro‐agents. (A) Conceptual illustration of electrospun fiber engineering showing fluorescent dye incorporation (Coumarin 6 and Nile Red), controllable fiber morphology (straight and beaded), and magnetic nanoparticle loading. (B) Fluorescence imaging of electrospun fibers with increasing dye loading in polystyrene (PS) (0.2–1.6%), imaged in the two detection channels (Channel 1, Channel 2). Top: Coumarin 6 (C6) fibers; bottom: Nile Red (NR) fibers. Corresponding plots show the 95th percentile (P95) fluorescence intensity versus dye concentration for each channel. (C) Straight and beaded fibers labeled with C6 or NR, illustrating morphology‐dependent fluorescence patterns. (D)Quantification of fluorescence intensity across dyes and morphologies. (E) Dual‐color imaging of fibers labeled with both dyes, showing raw and spectrally unmixed reconstructions for straight and beaded morphologies. (F) Fibers containing magnetic nanoparticles (${\mathrm{Fe}}_{3}O_{4}$) compared with non‐magnetic fibers. (G) Quantification of fluorescence intensity for fibers with and without ${\mathrm{Fe}}_{3}O_{4}$. (H) Scanning electron microscopy (SEM) images of straight and beaded fibers showing embedded magnetic nanoparticles (yellow arrows). All quantitative fluorescence data in panels (B), (D), and (G) are presented as mean ± SD across four FOVs (n=4). Scale bars = 50 μm (fluorescence) and 1 μm (SEM).

Electrospun fibers labeled with increasing dye loading (0.2–1.6 wt%) are shown in Figure 4B. For C6‐labeled fibers, Channel 1 captures the dominant fluorescence signal, while Channel 2 shows partial crosstalk due to spectral overlap. In contrast, NR‐labeled fibers emit predominantly in Channel 2 with negligible signal in Channel 1. Fluorescence intensity is quantified using the 95th percentile (P95) intensity. For C6, the signal increases approximately linearly over the investigated concentration range in both channels, indicating efficient dye incorporation with minimal self‐quenching. For NR, the signal also increases with dye loading, but the trend becomes sub‐linear at higher concentrations, suggesting concentration‐dependent quenching or aggregation within the polymer matrix. Based on these trends, the highest investigated dye loading (1.6 wt%) is selected for both C6 and NR in the subsequent analyses. The observed spectral overlap, particularly for C6, further motivates the later use of spectral unmixing for improved channel separation.

Morphology is further tuned to produce either straight fibers or fibers containing localized bead structures (Figure 4A). Representative two‐photon fluorescence images of straight and beaded fibers labeled with C6 and NR are shown in Figure 4C. Beaded fibers exhibit localized regions of increased fluorescence intensity for both dyes, consistent with dye accumulation within bead structures. Quantitative analysis confirms higher P95 fluorescence values for beaded fibers than for straight fibers (Figure 4D), reflecting the increased local fluorescent volume within bead regions. To improve spectral separation between the two fluorescent dyes, linear spectral unmixing is applied to the dual‐color images (Figure 4E). The unmixing procedure reduces C6 leakage into Channel 2 while preserving NR signal integrity, enabling clearer discrimination of spatially overlapping fluorescence signals.

Magnetic functionality is introduced by incorporating ${\mathrm{Fe}}_{3}O_{4}$ nanoparticles into the electrospinning solution while maintaining the overall solids concentration. Coumarin 6 is selected as the fluorescent dye for magnetic fibers because it provides a more stable two‐photon fluorescence response than Nile Red. Representative two‐photon images of fibers with and without ${\mathrm{Fe}}_{3}O_{4}$ are shown in Figure 4F. Incorporation of magnetic nanoparticles reduces fluorescence intensity (Figure 4G), which is attributed to partial replacement of fluorescent polymer by nanoparticles together with additional optical absorption and scattering introduced by ${\mathrm{Fe}}_{3}O_{4}$. Scanning electron microscopy images confirm the presence of embedded nanoparticles within both straight and beaded fibers while preserving the overall fiber morphology (Figure 4H).

### 2.3 Real‐Time Two‐Photon Imaging of Magnetically Actuated Micro‐Agents

Having established fluorescent labeling and magnetic loading of the precursor fibers, this section evaluates the ability of TPE to support reliable real‐time motion extraction from magnetically actuated micro‐agents. The analysis first considers ${\mathrm{Fe}}_{3}O_{4}$‐loaded magnetic micro‐agents labeled with Coumarin 6 (C6). In these single‐population experiments, the micro‐agents are derived from beaded electrospun fibers, suspended in a fluid‐filled chamber (75 μm in height), and actuated using a rotating permanent magnet positioned near the distal probe. Morphological analysis of magnetic micro‐agents yielded median long‐ and short‐axis dimensions of 18.1 μm (IQR: 14.9–19.6 μm) and 13.5 μm (IQR: 10.7–15.3 μm), respectively, with a median aspect ratio of 1.3 (IQR: 1.2–1.5) indicating a predominantly elongated morphology (Figure S6).

Magnetic actuation is generated using a diametrically magnetized permanent magnet mounted on a DC motor (Figure 5A). Magnet rotation produces a time‐varying magnetic field at the sample location, driving the reorientation and motion of the magnetic micro‐agents. The corresponding magnetic field trajectory at the workspace is shown in Figure 5B, where the field rotates in a plane with a magnitude of approximately 43–63 mT over each cycle (details in Section 4). From these measurements and the magnet specifications, the field gradient at the workspace is estimated to be on the order of 20 T $m^{-1}$, varying over each rotation cycle. In this rotating‐field regime, comparison of the magnetic torque scale with the gradient‐force contribution over a characteristic micro‐agent length indicates that torque‐driven reorientation dominates by approximately two orders of magnitude (Note S3). Figure S7 shows the experimental motor voltage‐to‐frequency mapping used to tune the actuation frequency. Figure S8 shows the measured temporal evolution of the magnetic field components and magnitude at the workspace. The actuation frequency is varied through the motor driving voltage, enabling experiments in the 0.5–2 Hz range.

**FIGURE 5:**
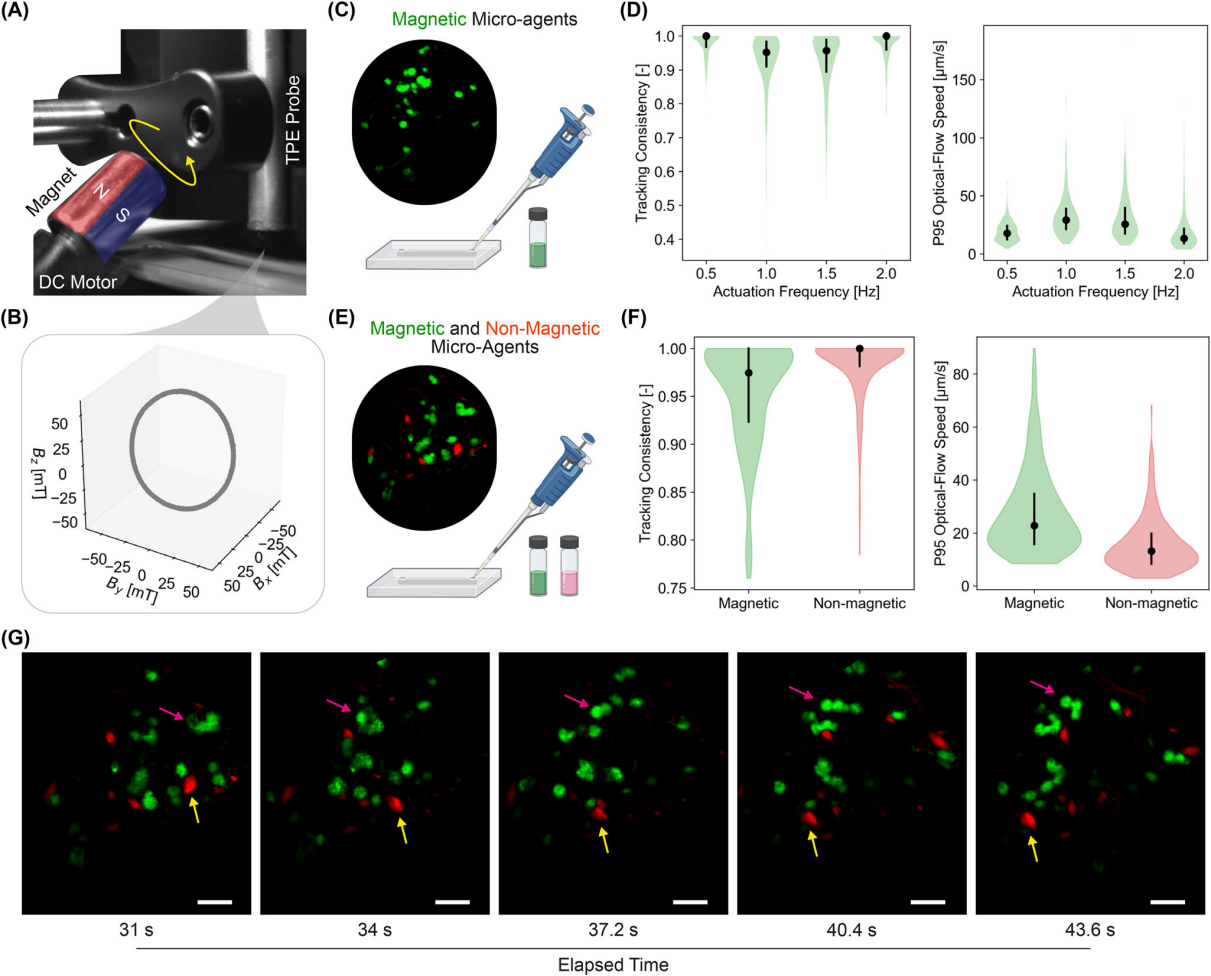
Magnetic actuation and dynamic motion analysis of fluorescent micro‐agents. (A) Experimental configuration for magnetic actuation of micro‐agents using a rotating permanent magnet mounted on a DC motor positioned near the distal TPE probe. (B) Magnetic field trajectory generated by the rotating magnet, illustrating the rotating magnetic field used for micro‐agent actuation. (C) Representative two‐photon image of magnetic micro‐agents (Coumarin 6, green) during actuation experiments. (D) Motion analysis for magnetic micro‐agents across actuation frequencies (0.5–2 Hz). Violin plots show the per‐frame tracking consistency (left) and P95 optical‐flow speed (right) at each frequency, with 350 per‐frame values per distribution; the marked point and bar give the median and interquartile range. (E) Representative dual‐color two‐photon image of magnetic (Coumarin 6‐labeled, green) and non‐magnetic (Nile Red‐labeled, red) micro‐agents. (F) Motion analysis for the dual‐color experiment, in which magnetic (Coumarin 6, green) and non‐magnetic (Nile Red, red) agents are actuated together at 1 Hz. Violin plots show the per‐frame tracking consistency (left) and P95 optical‐flow speed (right) for each population (250 frames each; median and interquartile range marked). (G) Representative time‐lapse sequence illustrating the motion of micro‐agents under magnetic actuation. Arrows indicate selected agents across frames. Scale bars = 50 μm. The complete dynamic sequences are provided in Video S1.

A representative TPE image of fluorescent magnetic micro‐agents, together with a schematic of the sample preparation, is shown in Figure 5C. Micro‐agent motion is quantified from the acquired image sequences using optical‐flow analysis. Consistent with the reduced fluorescence intensity observed after ${\mathrm{Fe}}_{3}O_{4}$ incorporation (Section 2.2), the excitation power is increased to 65 mW in the dynamic experiments to maintain a sufficient signal‐to‐noise ratio for reliable motion estimation.

Tracking consistency is quantified using a metric based on forward–backward optical‐flow agreement to assess the reliability of motion extraction from the acquired TPE sequences under magnetic actuation (details in Section 4). Median tracking consistency remains high (>0.9) across the tested actuation frequencies (Figure 5D, left), indicating stable feature tracking and consistent image quality during dynamic operation. The corresponding P95 optical‐flow speed distributions (Figure 5D, right) show a non‐monotonic dependence on actuation frequency. The median speed reaches 29.2 μm $s^{-1}$ at 1.0 Hz (IQR: 21.2–38.9 μm $s^{-1}$) and decreases to 13.3 μm $s^{-1}$ at 2.0 Hz (IQR: 9.1–21.7 μm $s^{-1}$). This roll‐off at higher frequency is consistent with the agents no longer following the rotating field in phase, indicating a frequency‐limited response under the tested conditions [14]. The broad distributions at each frequency further reflect heterogeneous, interaction‐mediated motion across the sample, consistent with local contacts and mechanical constraints within the chamber.

The analysis is then extended to mixed samples containing ${\mathrm{Fe}}_{3}O_{4}$‐loaded magnetic and ${\mathrm{Fe}}_{3}O_{4}$‐free non‐magnetic micro‐agents, labeled with Coumarin 6 and Nile Red, respectively. Dual color TPE is used to assess channel specificity and population‐specific motion analysis. The experiments are performed at 1 Hz, selected from the actuation sweep presented in Figure 5D, right. A representative dual‐color TPE image and the corresponding preparation schematic are shown in Figure 5E, where both populations can be distinguished. Quantitative analysis of the dual‐channel sequences shows high tracking consistency for both magnetic and non‐magnetic agents (Figure 5F, left). The same image sequences show that, although magnetic agents respond directly to the rotating magnetic field, nearby non‐magnetic agents also undergo displacement, likely due to local contact interactions and fluid‐mediated coupling within the chamber. Representative time‐lapse frames illustrating these interactions are shown in Figure 5G, where arrows identify selected magnetic and non‐magnetic agents across frames.

### 2.4 Two‐Photon Imaging of Magnetic Micro‐Agents Through Biological Tissue

To evaluate TPE performance under attenuated imaging conditions, magnetic micro‐agents are imaged through excised rat mammary gland tissue. Because ${\mathrm{Fe}}_{3}O_{4}$ incorporation reduces fluorescence intensity (Section 2.2), this experiment uses the magnetic agents as a stringent test of detectability through tissue. In this configuration, the tissue sample is placed in contact with the micro‐agents, while the TPE probe is positioned above the tissue surface so that fluorescence from the agents is detected through the scattering layer (Figure 6A). The tissue thickness in these experiments is approximately 140 μm (details in Section 4).

**FIGURE 6:**
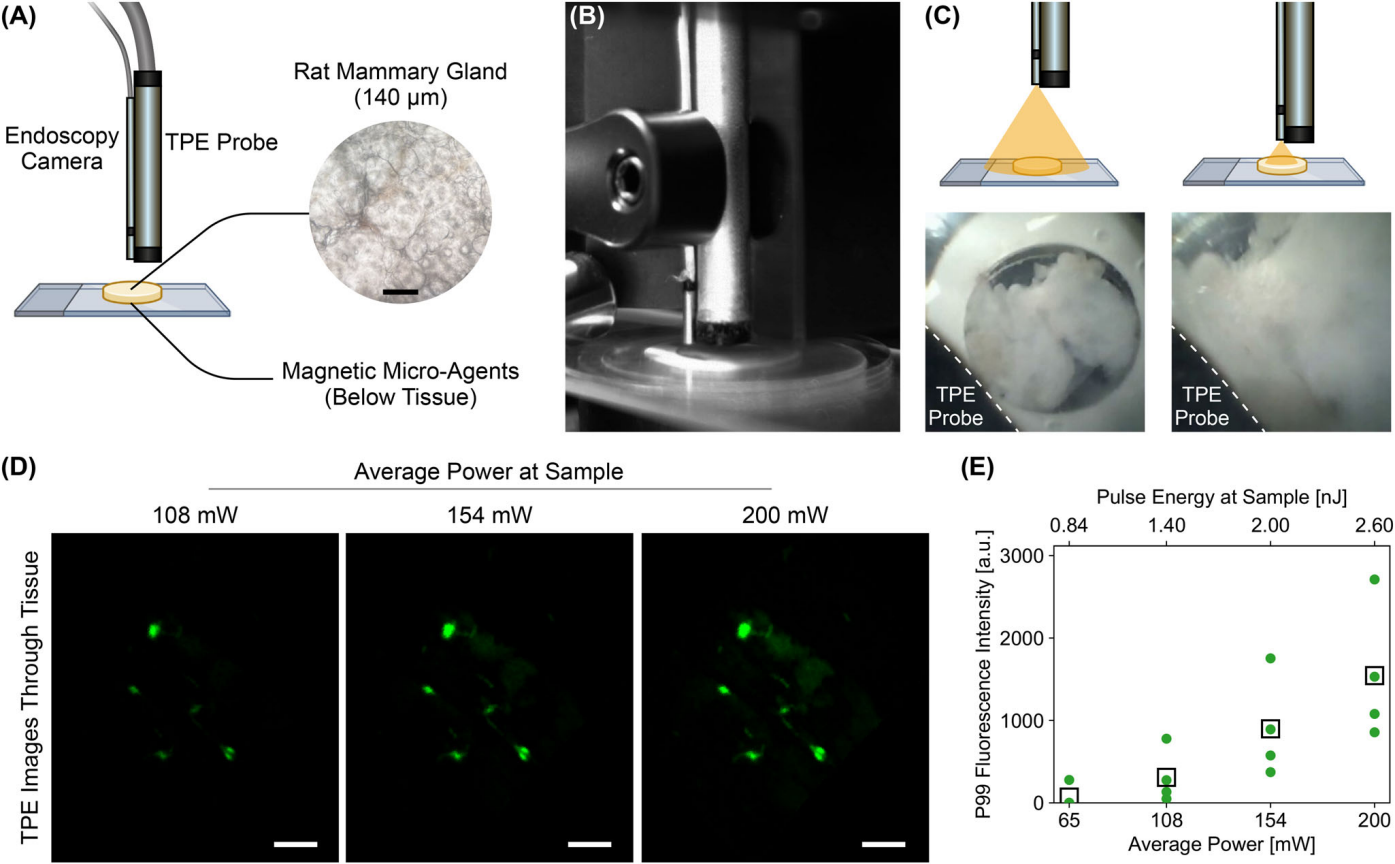
Two‐photon endomicroscopy (TPE) imaging of Coumarin 6‐labeled magnetic micro‐agents through biological tissue. (A) Conceptual schematic of the imaging configuration showing the TPE probe positioned above a rat mammary gland tissue sample (140 μm thickness), with fluorescent magnetic micro‐agents located beneath the tissue layer. An auxiliary endoscopic camera is positioned for surface visualization and probe alignment during the experiment. The inset shows a microscopy image of the rat tissue sample. Scale bar = 100 μm. (B) Experimental implementation of the imaging setup showing the distal TPE probe and endoscopic camera positioned above the tissue sample. (C) Representative endoscopic camera views illustrating probe positioning relative to the tissue sample during imaging experiments. The complete workflow is provided in Video S2. (D) TPE fluorescence images of magnetic micro‐agents acquired through the tissue layer at different average laser powers at the sample with a constant repetition rate (77 MHz). Scale bars = 50 μm. (E) Quantification of the 99th percentile (P99) fluorescence intensity as a function of average power at the sample, with the corresponding pulse energy on the top axis. Each measurement is averaged over 10 consecutive frames; individual measurements from four independent sample preparations (*n* = 4) are plotted as green circles, and squares denote the mean at each power level.

The experimental implementation of this configuration is shown in Figure 6B. A separate white‐light endoscopic camera attached to the TPE scanner is used to guide probe positioning relative to the tissue sample (Figure 6C). Video S2 shows the corresponding workflow, including surface positioning, axial scanning to identify the focal plane, and the excitation‐power sweep used for imaging through tissue.

Representative TPE fluorescence images acquired through the tissue layer at different excitation powers are shown in Figure 6D. Despite the attenuation introduced by the tissue, fluorescent magnetic micro‐agents remain detectable beneath the scattering interface, and the image intensity increases with excitation power while preserving spatial localization. The corresponding fluorescence intensity, quantified using the 99th percentile (P99) metric, increases with the average power delivered at the sample (Figure 6E), confirming the visual trend observed in the images.

Because through‐tissue imaging requires increased excitation power, illumination‐induced effects are assessed using a stationary‐beam control on Rhodamine B‐stained mammary gland tissue (Figure S9). The excitation beam is parked near the center of the tissue surface for 3 min at 240 mW, exceeding both the highest power used in the imaging experiments and the typical local exposure duration during scanning. Under these conditions, no visible change in tissue morphology is observed in the images, while the fluorescence intensity within the exposed region decreases by approximately 9.5% (Figure S9B–D).

To place this exposure in quantitative context, the onset of femtosecond laser ablation is commonly expressed as a fluence threshold, which depends on the tissue type and irradiation conditions [44]. The estimated per‐pulse fluence at the TPE probe focus is approximately 0.05 J ${\mathrm{cm}}^{-2}$ at the highest imaging power and 0.06 J ${\mathrm{cm}}^{-2}$ during the 240‐mW stationary‐beam control (calculation detailed in Section 4). These values are more than one order of magnitude below the reported 0.7 J ${\mathrm{cm}}^{-2}$ material‐removal threshold for bone [45] and below the 4.3 J ${\mathrm{cm}}^{-2}$ minimal‐visible‐lesion threshold reported for corneal neovascular tissue [46]. The fluence comparison and stationary‐beam control indicate no detectable gross structural alteration or ablation under the fixed‐tissue conditions used. This analysis specifically addresses structural damage and ablation. Photochemical phototoxicity and cumulative heating in viable tissue occur through distinct mechanisms at substantially lower exposures and are not quantified in this study.

Imaging micro‐agents beneath a tissue layer represents the intended operating regime of TPE, in which the endomicroscopic probe is positioned locally near a target site. Conventional fiber‐scanning TPE systems have demonstrated useful imaging depths on the order of 100–200 μm [40, 47, 48]. Recent probe designs have extended the accessible imaging depth, with enhanced fluorescence collection enabling two‐photon imaging to 300 μm in excised tissue and integrated focus tuning providing an axial imaging range of 400 μm over a 350 μm diameter field of view [49, 50]. In practice, actual penetration in tissue remains dependent on tissue attenuation, distal focusing geometry, and excitation and fluorescence collection efficiency [40].

Because ${\mathrm{Fe}}_{3}O_{4}$ incorporation reduces the emission fluorescence of the precursor fibers (Section 2.2), the practical detection depth of magnetic micro‐agents is expected toward the shallower end of the 100–200 μm regime typical of compact fiber‐scanning systems. Engineered micro‐agents with enhanced fluorescence contrast, together with improved detection sensitivity, could extend the accessible depth. In the present experiment, magnetic micro‐agents remain detectable through a 140 μm fixed mammary gland layer, consistent with the practical depth regime of conventional fiber‐scanning TPE. The fixed‐tissue configuration provides a controlled model for evaluating tissue‐induced attenuation while retaining aspects of tissue morphology and spatial heterogeneity. The absence of perfusion and physiological motion allows attenuation from the preserved tissue layer to be assessed independently, while these physiological processes remain relevant under in vivo conditions.

Within the broader landscape of deep‐tissue imaging, TPE occupies a local, near‐target regime, distinct from external modalities that provide greater penetration and spatial coverage. Clinical ultrasound provides label‐free, real‐time structural imaging at centimeter‐scale depths, with resolution and penetration determined by the acoustic frequency and imaging geometry [24, 51]. Photoacoustic computed tomography converts absorbed pulsed optical energy into acoustic waves detected by ultrasound transducers and has demonstrated whole‐body small‐animal and human breast imaging, including breast visualization at depths up to 4 cm [52, 53, 54]. Magnetic particle imaging directly detects the nonlinear magnetic response of superparamagnetic tracers and is not subject to signal attenuation by intervening tissue [28, 55]. These capabilities define complementary spatial regimes. External modalities support deep‐tissue visualization and organ‐scale localization, whereas TPE, through endoscopic access, provides high‐resolution imaging of a localized tissue volume.

At the local, near‐target scale, TPE is most directly compared with endoscopy and endomicroscopy modalities. For example, wide‐field fluorescence endoscopy provides real‐time, surface‐weighted fluorescence visualization but lacks depth‐resolved optical sectioning [56]. Confocal endomicroscopy provides cellular‐scale fluorescence optical sectioning over superficial depths and a submillimeter field of view [57]. Endoscopic optical coherence tomography provides label‐free, depth‐resolved structural imaging over millimeter‐scale depths, but does not provide fluorescence‐specific discrimination [58]. Photoacoustic endoscopy reaches greater, millimeter‐scale depths (1–3 mm) than TPE and can separate absorbers by their absorption spectra. However, this contrast is not fluorophore‐specific and is obtained at coarser spatial resolution [59]. Relative to these modalities, TPE combines nonlinear optical sectioning with micron‐scale, fluorophore‐specific multicolor contrast for distinguishing labeled micro‐agents from adjacent labeled biological structures. These modalities, together with representative external deep‐tissue systems, are compared in Table S1 across resolution, imaging depth, field of view, and other imaging parameters.

### 2.5 Image‐Guided Re‐Centering of a HeLa Cell Spheroid during Micro‐Agent Interaction

Beyond motion analysis and imaging through biological tissue, image feedback can also support active re‐centering during magnetic micro‐agent interaction. Maintaining a biological structure within the imaging field of view becomes important when contact with magnetic micro‐agents induces lateral displacement. Here, two‐photon endomicroscopy (TPE) is used to demonstrate the feasibility of image‐guided lateral re‐centering of a HeLa cell spheroid during magnetic micro‐agent interaction.

Image‐guided re‐centering is implemented through active probe repositioning to maintain the spheroid near the center of the TPE field of view. The experimental configuration shown in Figure 7A combines translational magnetic actuation with motorized stage correction. Magnetic micro‐agents and a spheroid with a diameter of approximately 200 μm are placed in a 190 μm‐high chamber, and the spheroid's motion is monitored in the lateral image plane. In contrast to the rotation‐based actuation experiments described in Section 2.3, a handheld permanent magnet is used to generate directional magnetic forces that translate the magnetic micro‐agents. As the agents interact mechanically with the spheroid boundary and induce lateral displacement within the chamber, motorized linear stages reposition the imaging probe relative to the sample along the x and y directions, thereby maintaining the spheroid near the center of the image.

**FIGURE 7:**
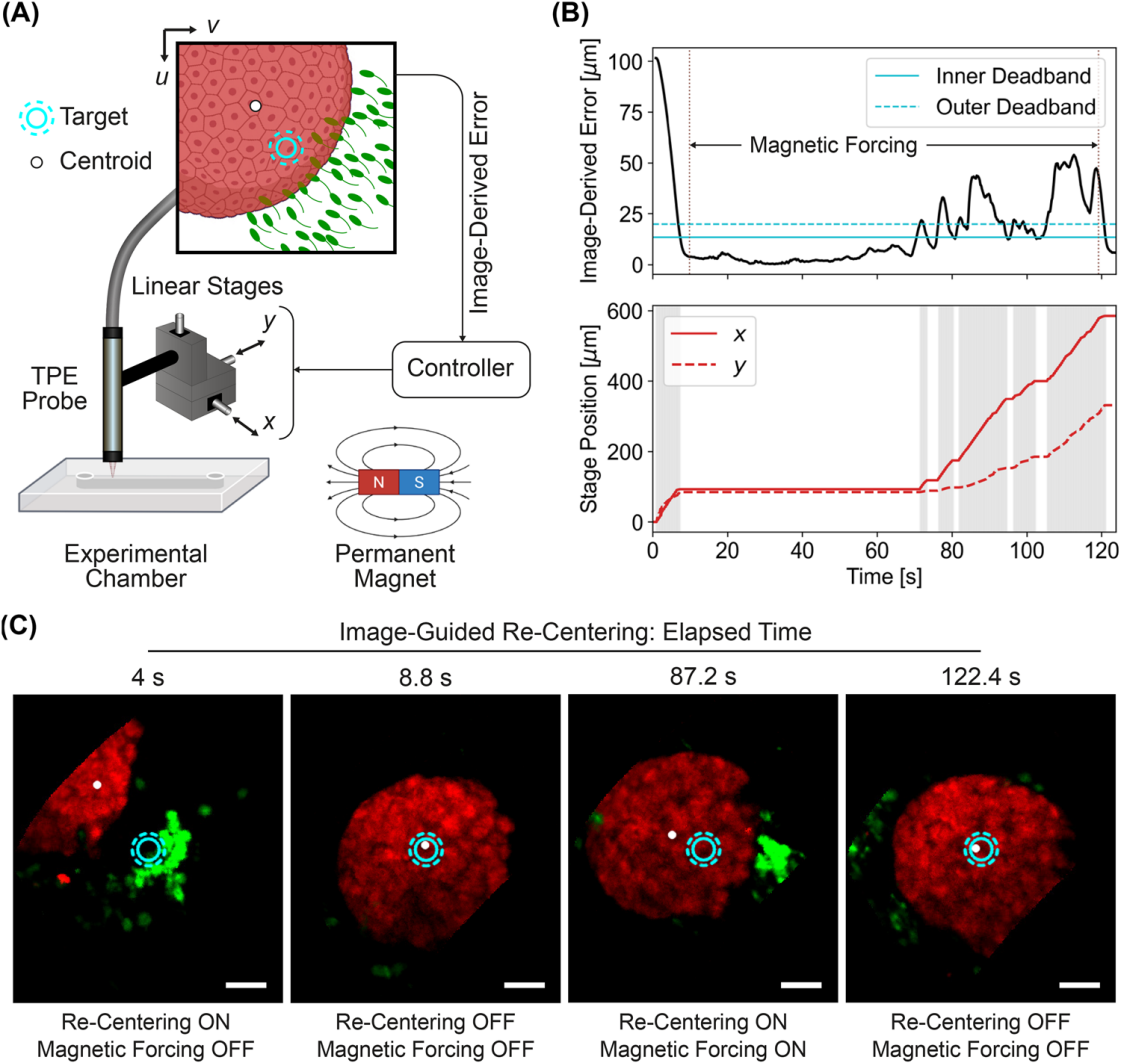
Image‐guided re‐centering using two‐photon endomicroscopy feedback. (A) Schematic of the image‐based feedback control system. The centroid of the target spheroid (Rhodamine B‐labeled, red) is extracted from the TPE image and compared with a predefined target location in the field of view. A controller uses the resulting image‐derived error to command motorized linear stages that adjust the probe position relative to the sample while magnetic micro‐agents (Coumarin 6‐labeled, green) induce target displacement. (B) Re‐centering performance. Top: image‐derived error relative to the target location, with the inner and outer deadband thresholds indicated. Magnetic forcing is applied intermittently from *t* = 10.0 to 119.0 s. Bottom: corresponding relative stage positions along the x and y directions. The grey regions indicate periods during which stage correction is active. (C) Representative dual‐color TPE frames acquired during the re‐centering experiment at different elapsed times. The cyan marker indicates the target location, and the white dot represents the detected spheroid centroid. Magnetic micro‐agents (green) displace the spheroid through direct mechanical pushing, while closed‐loop control restores its image position within the inner deadband. Scale bars: 50 μm. The complete dynamic sequence is provided in Video S3.

The spheroid position is estimated in real time by segmenting its visible region within the TPE field of view and computing the centroid of the resulting mask. The distance between this centroid and a predefined target position defines the image‐derived error used as the control variable. Figure 7B, top shows the corresponding image‐derived error over time. To limit stage oscillations while maintaining centering accuracy, inner and outer deadbands are applied. When the error exceeds the outer deadband (20.0 μm), stage correction is triggered; correction stops once the error returns within the inner deadband (13.3 μm). The corresponding relative stage positions along the x and y axes are shown in Figure 7B, bottom.

Because the magnetic forcing is applied manually with a handheld permanent magnet, the induced spheroid displacements occur intermittently and with variable magnitudes and directions. Re‐centering performance is therefore evaluated event‐wise, with each outward crossing of the outer deadband defining a perturbation event. Following the onset of magnetic forcing (t = 10.0 s), seven perturbation events are detected within the analyzed actuation sequence. Each event is followed by a return of the image‐derived error to the inner deadband, with a median recovery time of 6.0 s (IQR: 3.9–11.7 s). During magnetic forcing, the largest tracked error excursion followed by recovery is approximately 54 μm. Over the analyzed sequence, the relative stage positions span 585 and 331 μm along the x and y axes, respectively (Figure 7B).

Representative dual‐color TPE frames at selected time points are shown in Figure 7C, capturing the progressive re‐centering of the spheroid. Magnetic micro‐agents (green) accumulate near the spheroid boundary, and the spheroid is displaced through direct mechanical pushing. Together, these frames illustrate image‐guided re‐centering of a HeLa cell spheroid while preserving dual‐color visualization of the interacting magnetic micro‐agents.

Previous studies on photoacoustic‐ and ultrasound‐guided microrobotic systems have demonstrated navigation over larger imaging regions and at greater tissue depths [60, 61]. In comparison, the present TPE approach provides local, high‐resolution fluorescence feedback near the micro‐agent–target interaction site, but with a smaller field of view and limited penetration depth. Accordingly, the TPE‐guided re‐centering demonstrated here represents a near‐target feedback strategy complementary to modalities intended for wider‐range navigation.

## 3 Conclusions and Future Work

This study demonstrates a two‐photon endomicroscopy (TPE) system for real‐time fluorescence imaging of magnetically actuated micro‐agents. The TPE system enables image‐based motion analysis, while dual‐color imaging expands its capability to distinguish mixed micro‐agent populations labeled with distinct fluorescent dyes. Magnetic micro‐agents remain detectable through a 140 μm fixed mammary gland tissue layer. Real‐time visual feedback further supports a feasibility demonstration of image‐guided re‐centering, keeping a displaced HeLa cell spheroid within the field of view during magnetic micro‐agent interaction. These results extend TPE from passive observation to active image‐guided functionality in microrobotic systems. Future work will extend imaging to living tissue, where heterogeneity, perfusion, and physiological motion become relevant. Additionally, a new fabrication approach for the magnetic micro‐agents will be explored to lower the excitation power required for imaging. Further directions include multimodal imaging, reproducible magnetic perturbations for systematic evaluation of closed‐loop re‐centering, and advanced control strategies for coordinated guidance of multiple micro‐agents. Such developments can further expand the role of endomicroscopy in minimally invasive microrobotic interventions.

## 4 Experimental Section

### 4.1 Two‐Photon Endomicroscopy System

The TPE system employed a wavelength‐tunable femtosecond pulsed laser (Cronus 2P, Light Conversion, Lithuania) operating at 900 nm with a spectral bandwidth of 9 nm. The laser delivered pulses of 160 fs duration at a repetition rate of 77 MHz with a beam diameter of approximately 3 mm. Pulse chirp was adjusted using the internal compressor to compensate for dispersion introduced by downstream optical components.

The excitation beam was coupled into the core of a 0.65 m double‐clad fiber (DCF) (IXF‐2CF‐PAS‐4‐130‐0.14, iXblue, France) using an infinity‐corrected microscope objective (EC Epiplan‐NEOFLUAR 20×/0.5 HD, Leica Microsystems GmbH, Germany). The DCF was routed concentrically through a lead–zirconate–titanate (PZT) tube scanner (PIC255, PI Ceramic GmbH, Germany), enabling lateral scanning of the fiber tip.

The fiber was supported within the scanner using two custom adapters fabricated from IP‐S photoresin (Nanoscribe GmbH, Germany) via dip‐in laser lithography (Photonic Professional GT2, 10×/0.3 NA objective). The adapters were bonded to the PZT tube using cyanoacrylate adhesive (Loctite 401, Henkel AG & Co. KGaA, Germany). The DCF ends were cleaved prior to assembly and bonded to the adapters using UV‐curable optical adhesive (NOA68, Norland Products, USA). A protective fiber jacket terminated at the proximal adapter and was fixed using cyanoacrylate adhesive to provide mechanical stability while allowing the distal fiber segment to oscillate freely.

The PZT–DCF assembly was housed within an aluminum probe body using a laser‐cut acrylic support (Speedy 400, Trotec Laser GmbH, Austria). At the distal end of the probe, a gradient‐index (GRIN) lens (#64‐538, Edmund Optics Inc., USA) was mounted in a dedicated acrylic holder. The holder was bonded to the probe housing using cyanoacrylate adhesive, while the GRIN lens was fixed using optical adhesive (NOA63, Norland Products, USA) to ensure stable optical alignment. The axial spacing between the fiber tip and GRIN lens was adjusted using a digital caliper (500‐196‐30, Mitutoyo Corp., Japan).

During imaging, excitation light emerging from the DCF core was focused onto the sample by the distal GRIN lens. Fluorescence generated at the focal region was collected by the inner cladding of the DCF and relayed back through the proximal objective to the detection optics. The returning emission was separated into two spectral channels using dichroic mirrors (DMLP805 and DMLP605R, Thorlabs, USA) and bandpass filters (HQ510/20m and ET645/75m, Chroma, USA), corresponding to wavelength ranges of 500–520 nm (channel 1) and 608–682 nm (channel 2). The filtered signals were focused by collection lenses (AC254‐150‐A‐ML, Thorlabs, USA) onto photomultiplier tubes (PMT1001/M and PMT2101/M, Thorlabs, USA). A summary of the transmission curves for dichroic mirrors and bandpass filters is presented in Figure S1.

### 4.2 Scanning and Image Formation

Lateral scanning of the distal fiber tip was achieved by driving the four electrodes of the PZT tube scanner using a voltage amplifier (TD250‐INV, PiezoDrive, USA) controlled by a function generator (33510B, Keysight, USA). The 10‐MHz reference clock generated by the function generator was shared with a two‐channel high‐speed digitizer (ATS‐9146, AlazarTech, Canada), ensuring a common timing base for both waveform generation and acquisition. In addition, a hardware trigger derived from the function generator was used to initiate digitizer acquisition, providing deterministic synchronization between the scanner drive signals and fluorescence detection in two spectral channels.

Fluorescence signals from the two photomultipliers were digitized at 16‐bit resolution and 10 ${\mathrm{MS}}^{-1}$ sampling rate using the synchronized digitizer. The digitizer was operated with full analog bandwidth (no bandwidth limiting enabled). Image formation was performed online in real time (5 fps) using pre‐calibrated lookup tables that map the instantaneous fiber‐tip position to the acquired waveform samples. Because Lissajous scanning produced a non‐uniform dwell‐time distribution across the valid scanned region, image intensities were normalized by the local sampling density during reconstruction. For the 300 × 343 px reconstruction grid enclosing the scan trace, the median dwell time across valid pixels was 2.6 μs, with a 5th–95th percentile range of 1.1–8.6 μs at 5 fps and 10 ${\mathrm{MS}}^{-1}$ sampling rate. The lookup tables were generated from measurements obtained with a lateral beam‐position sensor (PDP90A, Thorlabs, USA) placed downstream of the probe. The calibration signals were acquired using the same digitizer and timing configuration as used during imaging, ensuring consistent temporal registration. During image formation, the non‐uniform pixel sampling inherent to the Lissajous scan was compensated by normalizing the accumulated signal by the corresponding dwell time at each valid pixel. During routine operation, the acquisition bandwidth results in a sustained data throughput of approximately 40 MB $s^{-1}$.

### 4.3 Micro‐Agent Fabrication:

Electrospun fluorescent fibers were first fabricated and subsequently fragmented via ultrasonic treatment into discrete, micrometer‐scale fiber fragments, referred to here as micro‐agents. The term was used operationally: these agents were functionalized only for magnetic actuation and fluorescence imaging. Micro‐agents prepared without iron oxide (${\mathrm{Fe}}_{3}O_{4}$) nanoparticles were designated non‐magnetic and were not directly actuated by the applied magnetic field, whereas those incorporating ${\mathrm{Fe}}_{3}O_{4}$ were designated magnetic. Polystyrene (PS) pellets (430102–1KG, Sigma‐Aldrich, USA) were used as the carrier polymer and were dissolved in anhydrous N,N‐dimethylformamide (DMF) (227056–1L, Sigma‐Aldrich, USA). For fluorescence labeling, Coumarin 6 (442631–1G, Sigma‐Aldrich, USA) or Nile Red (72485, Sigma‐Aldrich, USA) was incorporated into the polymer solution.

For non‐magnetic fibers used in fluorescence characterization experiments, PS solutions with total polymer concentrations of 28 and 45 wt% in DMF were prepared. The dye concentration was varied according to the desired loading and expressed relative to the PS mass fraction. Each solution was homogenized by stirring for 24 h using a roller mixer (LLG‐uniRoller 6, LLG‐Labware, Germany).

Electrospinning was performed by loading the polymer solution into a plastic syringe connected through Teflon tubing to an 18 gauge blunt‐tip needle (TS18SS‐15, Adhesive Dispensing Ltd., UK). A voltage of 14 kV was applied between the needle and a grounded collector, with a feed rate of 2.5 mL $h^{-1}$ and a tip‐to‐collector distance of 16 cm. Randomly oriented fiber meshes were collected on grounded aluminum foil under ambient conditions (20°C, 30% relative humidity) and dried at room temperature for 12 h to remove residual solvent.

For magnetic fiber fabrication, ${\mathrm{Fe}}_{3}O_{4}$ nanoparticles (637106–25G, Sigma‐Aldrich, USA) were incorporated into the electrospinning solution. The total solids concentration was maintained at either 28 or 45 wt%, while PS and ${\mathrm{Fe}}_{3}O_{4}$ were mixed at a mass ratio of 2:1. Thus, ${\mathrm{Fe}}_{3}O_{4}$ partially replaces PS relative to the corresponding non‐magnetic formulation while the total solids concentration remains constant. Coumarin 6 was used as the fluorescent dye for magnetic fibers at a concentration of 1.6 wt% relative to the total solids.

To generate micro‐agents, the deposited fiber meshes were cut into 2–3 mm pieces and transferred to a glass vial containing 1% (v/v) Tween 80 (P4780‐100ML, Sigma‐Aldrich, USA) in Milli‐Q water. The fibers were fragmented using an ultrasonic bath (Model 2510, Branson, USA) for 2 h, producing discrete fluorescent micro‐agents. Non‐magnetic micro‐agents were allowed to sediment naturally for approximately 2 h, whereas magnetic micro‐agents were collected using a permanent magnet. In both cases, the supernatant was removed and replaced with fresh Milli‐Q water containing 1% Tween 80.

### 4.4 Dispersion Compensation and Optical Power Measurements:

Group delay dispersion (GDD) precompensation was adjusted using the internal compressor of the femtosecond laser (Cronus 2P, Light Conversion, Lithuania) to optimize pulse delivery at the distal probe tip. For this characterization, straight electrospun fluorescent fibers were used as test samples. The dispersion setting was varied from no precompensation (0 ${\mathrm{fs}}^{2}$) to the maximum negative value available from the integrated compressor (−20000 ${\mathrm{fs}}^{2}$). For each dispersion setting, ten consecutive fluorescence images were acquired at 16‐bit depth, and fluorescence intensity statistics were computed from the resulting image sequences (Figure S5A,B). To validate the dispersion settings used during imaging experiments, second‐harmonic generation (SHG) signals were measured using a microscope‐slide peak‐power sensor (NS170C, Thorlabs, USA) (Figure S5C).

The average excitation power delivered at the probe output was measured using a silicon photodiode sensor (S130C, Thorlabs, USA). Both sensors were connected to the same power meter console (PM400, Thorlabs, USA), and all measurements were averaged over 100 samples for each experimental condition. During electrospun fiber characterization, the average excitation power at the sample plane was maintained at 16 mW. For dynamic imaging experiments, the average power was increased to 65 mW to maintain a sufficient fluorescence signal for motion analysis.

### 4.5 Rat Tissue Sample:

The rat mammary gland used in this study was collected post‐mortem from outbred albino naïve female rats (RjHan: WI, Janvier Labs, France). Organs and biological samples were prepared and disposed of according to the Cat.1 directive for animal bio‐products. The organs were fixed overnight in a 10% buffered formalin solution (Formalin 10%, Qpath Ref: 11699404, VWR). Subsequently, the tissues were immersed in 100% ethanol for 24 h and then stored at 4°C. The fixed mammary gland was carefully sliced with a scalpel and placed between two coverslips for surface flattening, together with a custom spacer made of plastic film with double‐sided medical‐grade adhesive (ARcare 90106NB, Adhesives Research, Inc., USA). The spacer, approximately 140 μm thick, was fabricated by laser cutting (Speedy 400, Trotec Laser GmbH, Austria), forming a ring with an 8 mm inner diameter. To enable imaging through rat tissue, 2 μL of micro‐agent solution was deposited within the inner ring and allowed to dry at ambient temperature before placing the rat tissue on top. Finally, 2 μL of Dulbecco's phosphate‐buffered saline was added to maintain a humid environment during the experiments.

### 4.6 Auxiliary Endoscopic Imaging:

An auxiliary endoscopic camera (V1000LH, Misumi Electronics Corp., Japan) was used to visualize the tissue surface during tissue imaging experiments. The camera was positioned adjacent to the TPE probe and provides wide‐field views of the tissue surface for probe alignment and positioning.

### 4.7 Cell Culture and Spheroid Preparation:

HeLa cells were cultured in Dulbecco's Modified Eagle Medium (11–965‐092, Fisher Scientific Ltd., Canada) supplemented with 10% fetal bovine serum (F7524–500ML, Sigma–Aldrich, USA) and 1% penicillin–streptomycin (15–140‐122, Thermo Fisher Scientific Inc., USA). Cells were maintained at 37°C in a humidified incubator with 5% ${\mathrm{CO}}_{2}$.

For spheroid formation, cells were trypsinized at approximately 80% confluency, counted, and resuspended in culture medium at a concentration of $2\times 10^{6}$ cells ${\mathrm{mL}}^{-1}$. A volume of 0.2 mL of the suspension was seeded onto an agarose microwell mold containing 1500 wells with a diameter and depth of 200 μm. Cells self‐aggregate within the microwells and form compact spheroids after approximately 3 days of incubation.

Prior to imaging experiments, spheroids were stained using a 1 mM Rhodamine B solution prepared in phosphate‐buffered saline to enhance fluorescence contrast. The stained spheroids were subsequently rinsed with fresh buffer and fixed for 15 min in 4% formaldehyde (F8775–25ML, Sigma‐Aldrich, USA) at room temperature. After fixation, the spheroids were washed twice with Dulbecco's phosphate‐buffered saline before being introduced into the experimental chamber for image‐guided re‐centering experiments.

### 4.8 Spectrum Analysis:

The emission spectra of fluorescent dyes used for micro‐agent fabrication were acquired using a spectrofluorometer (FP‐8300, Jasco, Japan) with a 1 nm data interval. For Coumarin 6 and Nile Red samples preparation, we dissolved 1.5 μg dye in 700 μL of dimethylformamide (DMF) (227056–1L, Sigma‐Aldrich, USA). Both solutions were blended for 2 h using a roller mixer (LLG‐uniRoller 6, LLG‐Labware, Germany). For analysis, the blends were placed in quartz glass cuvettes (CV10Q700F, Thorlabs, USA). The emission plots are presented in Figure S1. These spectra were used as an initial reference for dye selection and TPE system design. The corresponding fluorescence performance was evaluated experimentally under two‐photon excitation (Section 2.2).

### 4.9 Tissue Staining:

To enable fluorescence visualization of the tissue surface and evaluate potential illumination‐induced structural changes, rat mammary gland samples were stained with 2 μL of a 1 mM Rhodamine B solution prepared in phosphate‐buffered saline.

### 4.10 Magnetic Actuation:

Magnetic actuation was implemented using permanent magnets to generate rotating or translational magnetic fields at the sample location. For rotational actuation experiments, a diametrically magnetized NdFeB ring magnet (R06.8×03×10GD‐40SH, Article No. 9963‐6098, HKCM Engineering GmbH, Germany) was mechanically coupled to a DC gearmotor (2358, Pololu Corp., USA). The motor was powered using a programmable power supply (E36313A, Keysight Technologies, USA), allowing control of the magnet rotation frequency. The magnetic field at the sample workspace was characterized with a high precision three‐axis teslameter probe (3MH3A‐500MT, Senis AG, Baar, Switzerland). A dipole‐based estimate of the corresponding field gradient is provided in the Supporting Information. For image‐guided re‐centering experiments, a handheld neodymium–iron–boron (NdFeB) permanent magnet (S‐45‐30‐N, Supermagnete, Germany) was used to generate directional magnetic forces on the micro‐agents.

### 4.11 Statistical Analysis:

For fluorescence‐characterization experiments, each field‐of‐view (FOV) value was obtained by averaging four consecutive frames acquired at 5 fps (technical repeats used to reduce frame‐to‐frame noise, not independent observations), unless a different number was stated in the caption. Group data for these experiments are reported as mean ± standard deviation across the stated unit, which was the number of FOVs for fiber characterization and the number of independent sample preparations for the through‐tissue quantification (n specified per figure). Morphology and re‐centering results were reported as the median and interquartile range (IQR), over individual micro‐agents (*n* = 56) from 10 independent frames and over perturbation events within a single actuation sequence (*n* = 7), respectively. Optical‐flow speed and tracking consistency were reported as distributions of per‐frame values over the actuation phase, summarized by the median and IQR (Figure 5). These values come from consecutive frames of a single continuous recording and were therefore not treated as independent measurements.

### 4.12 Magnetic Micro‐Agent Morphology Analysis:

Magnetic micro‐agents were segmented from the C6 fluorescence channel after Gaussian smoothing using Otsu‐derived threshold. Small image artifacts were removed, and touching objects were separated using watershed segmentation. All detected regions were subsequently inspected manually to retain individual micro‐agents and exclude fiber remnants, unresolved agglomerates, and poorly segmented objects. The long and short axes and projected area were obtained from each accepted segmented region, and the aspect ratio was calculated as the long axis divided by the short axis. Measurements were converted to physical units using an image scale of 1.11 μm ${\mathrm{pixel}}^{-1}$.

### 4.13 Optical Flow–Based Motion Analysis:

Micro‐agent motion was quantified using feature‐based optical‐flow analysis implemented in Python with OpenCV. Image sequences acquired during magnetic actuation were processed frame by frame to estimate local displacement vectors between consecutive frames using the pyramidal Lucas–Kanade method [62]. Feature points were detected with the Shi–Tomasi corner detector [63] and tracked between consecutive frames to estimate local displacement vectors.

Two quantitative metrics were computed from the optical‐flow analysis: the optical‐flow speed and the tracking consistency. The optical‐flow speed is defined as the 95th percentile of the displacement‐magnitude distribution for each frame, providing a robust descriptor of the upper range of observed motion. Pixel displacements were converted to physical units using the spatial calibration of the imaging system and multiplied by the frame rate (5 fps) to obtain speeds in μm $s^{-1}$. Tracking consistency was used to assess motion‐estimation reliability through a forward‐backward consistency check. Feature positions tracked forward between consecutive frames are subsequently tracked backward to the original frame, and the Euclidean distance between the original and reconstructed positions was computed. Vectors with forward–backward error below 1 pixel were considered consistent, and tracking consistency is defined as the fraction of consistent vectors within each frame. All metrics were computed during the actuation phase and summarized across frames using the median and interquartile range for each actuation frequency.

### 4.14 Image‐Guided Re‐Centering and Stage Actuation:

Image‐guided re‐centering was implemented through closed‐loop visual servo control using DC servo motor actuators (Z812B, Thorlabs, USA) to reposition the imaging probe along the x and y directions. Each actuator was driven by a T‐Cube DC servo controller (TDC001, Thorlabs, USA). The controllers were mounted on a T‐Cube controller hub (TCH002, Thorlabs, USA), which provided power distribution and a USB interface for simultaneous control through a single connection to the host computer. Stage motion was commanded through the Kinesis control interface with a maximum velocity of 0.05 mm $s^{-1}$ and an acceleration of 0.5 mm $s^{-2}$ to ensure smooth probe motion during imaging.

The spheroid position was estimated directly from the TPE image stream by segmenting the spheroid fluorescence signal in each frame after Gaussian smoothing, intensity thresholding, and morphological filtering. The centroid of the largest segmented region was used to compute the image‐derived error relative to a predefined target location at the center of the field of view. The image‐derived error $\left(e_{x},e_{y}\right)$ was converted into stage commands using a proportional control law

$$\Delta x=-K_{p}e_{x}p,\;\Delta y=-K_{p}e_{y}p,$$

where p is the effective pixel size (1.11 μm ${\mathrm{pixel}}^{-1}$) and $K_{p}$ is the proportional gain. To reduce oscillatory corrections, a hysteresis‐based deadband strategy is applied: stage motion is triggered only when the error exceeds an outer deadband and stops once it returns within an inner deadband.

### 4.15 Optical Beam Characterization:

The focal spot size of the TPE probe was measured using an auxiliary optical imaging setup. The emitted beam was imaged directly onto a CMOS camera (acA2040‐90um, Basler AG, Germany) using a finite‐corrected microscope objective (100×, Mitutoyo 375‐053, Mitutoyo Corp., Japan). A neutral density filter (NE10A‐B, Thorlabs, USA) was placed in the optical path to attenuate the beam during these measurements. To estimate the effective numerical aperture of the probe, beam propagation measurements were performed using the same camera with the bare sensor exposed to the beam.

### 4.16 Experimental Chamber Fabrication:

Experimental imaging chambers were fabricated using standard glass microscope slides as the base substrate. Circular chambers with an 8 mm diameter were created by laser cutting adhesive spacer films using a ${\mathrm{CO}}_{2}$ laser system (Speedy 400, Trotec Laser GmbH, Austria). Two spacer materials were used depending on the experiment. A transfer adhesive film (3M 9969, 3M, USA) providing a chamber height of approximately 75 μm was used for magnetic micro‐agent dynamic analysis. For spheroid re‐centering experiments, a thicker medical‐grade adhesive spacer (ARcare 90106NB, Adhesives Research Inc., USA) with a thickness of approximately 190 μm was used to accommodate the larger sample volume. The adhesive spacers were applied onto the glass slide to define the chamber boundary, and the chambers were sealed using a glass coverslip placed on top of the spacer to enclose the sample.

### 4.17 Numerical Simulations:

Numerical simulations were performed to support the design and characterization of the scanning probe and optical focusing performance. The mechanical behavior of the fiber scanner inside the piezoelectric tube actuator was analyzed using finite‐element simulations in COMSOL Multiphysics (COMSOL AB, Sweden). The simulations were used to estimate the resonance behavior and lateral displacement of the oscillating fiber under sinusoidal excitation.

Optical propagation and distal focusing were analyzed in OpticStudio (Zemax LLC, USA) using physical‐optics simulations. The model includes the double‐clad fiber output and the gradient‐index (GRIN) lens, and examines both centered and decentered input conditions to evaluate beam propagation, focal spot size, and the effective numerical aperture at the sample plane.

### 4.18 Linear Spectral Unmixing:

Linear spectral unmixing was used to reduce fluorescence crosstalk between the two detection channels. The measured intensity in Channel 2 ($I_{2}$) was assumed to contain the desired Channel 2 signal ($I_{2,\mathrm{true}}$) and a contribution from the measured Channel 1 intensity ($I_{1}$):

$$I_{2}=I_{2,\mathrm{true}}+\alpha I_{1},$$

where the crosstalk coefficient (α) describes how much of the Channel 1 signal is detected in Channel 2.

For each imaging configuration, the crosstalk coefficient (α) was determined using a single‐color control acquired with the same sample composition, excitation power, and detector settings as the corresponding dual‐color experiment. After background subtraction, the coefficient is calculated as

$$\alpha =\frac{\sum_{p\in \Omega }I_{1,p}I_{2,p}}{\sum_{p\in \Omega }I_{1,p}^{2}},$$

where the pixel position (p) identifies the same location in both channels, the calibration mask (Ω) contains fluorescent and non‐saturated pixels, and the pixel intensities in Channels 1 and 2 are denoted by $I_{1,p}$ and $I_{2,p}$, respectively.

Each calibration used eight fields of view (n=8). Four consecutive frames were acquired at 5 fps in each field of view to reduce frame‐to‐frame noise. The coefficient was calculated for every frame, after which the median of the four values was used for that field of view. The median across the eight fields of view was then used as the crosstalk coefficient for the corresponding experiment.

The corrected Channel 2 intensity ($I_{2,\mathrm{corr}}$) is calculated as

$$I_{2,\mathrm{corr}}=\max\left(I_{2}-\alpha I_{1},\;0\right),$$

with negative values set to zero. The dual‐population and re‐centering experiments used the same Coumarin 6‐labeled magnetic micro‐agents under matched excitation power and detector settings, so the coefficient α=2.15 determined for the dual‐population experiment was applied directly to the re‐centering experiment. The differing Channel 2 species (Nile Red for the non‐magnetic agents, Rhodamine B for the spheroid) does not affect α, since neither contributes to Channel 1 leakage. A value greater than one reflects the different detector responses and gains of the two channels. Single‐color Nile Red and Rhodamine B controls show no measurable crosstalk into Channel 1, so no correction is applied in the reverse direction.

## Conflicts of Interest

The authors declare no conflicts of interest.

## Supporting information


**Supporting File 1**:smll75051‐sup‐0001‐SuppMat.pdf.

**Supporting File 2**: smll75051‐sup‐0002‐VideoS1.mp4.

**Supporting File 3**: smll75051‐sup‐0003‐VideoS2.mp4.

**Supporting File 4**: smll75051‐sup‐0004‐VideoS3.mp4.

## Data Availability

The data that support the findings of this study are available from the corresponding author upon reasonable request.
