# Supplementary material for: Two‐Photon Endomicroscopy for Image‐Guided Magnetic Micro‐Agents
Source: Small. 2026 Aug 11;22(56):e75051. doi: 10.1002/smll.75051 (PMC13653355; doi:10.1002/smll.75051)
Supplement: Supplementary file 1 [file SMLL-22-e75051-s001.pdf]

Supplementary Information for:

Two-Photon Endomicroscopy for Image-Guided Magnetic Micro-Agents

Juan J. Huaroto, Gert-willem Römer, Ivo M. Vellekoop, Sarthak Misra**

Juan J. Huaroto and Prof. Sarthak Misra
Surgical Robotics Laboratory, Department of Biomechanical Engineering, University of Twente, 7522 NB, Enschede, The Netherlands.
*Corresponding Authors: j.j.j.huarotosevilla@utwente.nl, s.misra@utwente.nl

Prof. Gert-willem Römer
Chair of Laser Processing, Department of Mechanics of Solids, Surfaces & Systems (MS3), University of Twente, 7522 NB The Netherlands.

Prof. Ivo M. Vellekoop
Biomedical Photonic Imaging Group, Faculty of Science and Technology, University of Twente, 7522 NB The Netherlands.

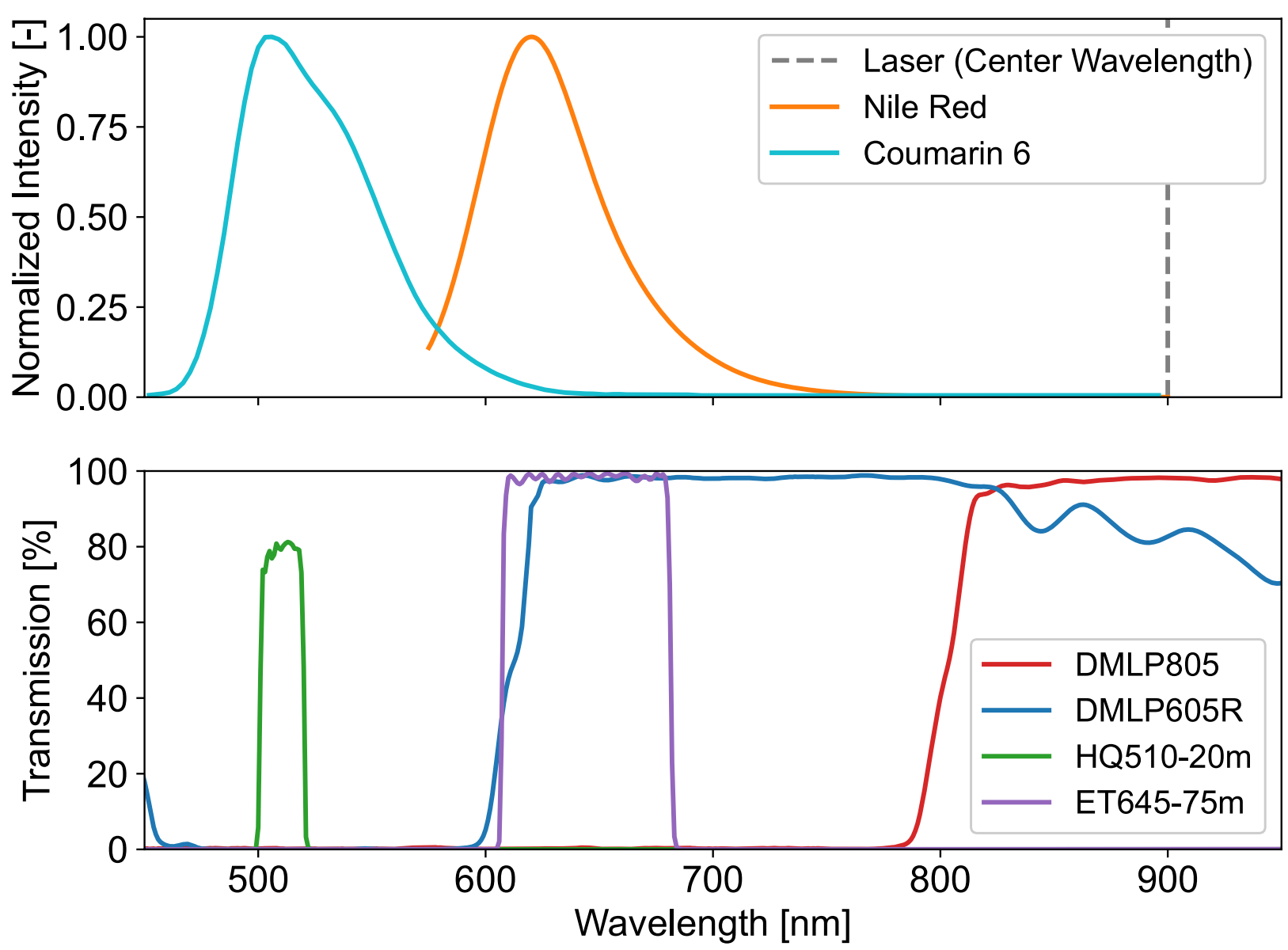

Figure S1: Spectral reference for dye selection and channel separation in the TPE system. Top panel: Normalized one-photon emission spectra of Coumarin 6 and Nile Red acquired using a spectrofluorometer. The dashed vertical line marks the 900 nm excitation wavelength used in the microscopy system and is shown only as a reference to the TPE excitation source. Spectra are normalized to their respective maxima for visualization. Bottom panel: Transmission spectra of the dichroic mirrors (DMLP805, DMLP605R) and bandpass filters (HQ510-20m, ET645-75m) used in the excitation and detection paths. These plots summarize the spectral rationale used for dye selection and nominal channel separation.

Note S1: Optical Design of the Distal Assembly

The distal optical response of the probe is analyzed using physical-optics simulations (Zemax LLC, USA) of the double-clad fiber (DCF) coupled to the GRIN lens. The main geometric control parameter is the axial spacing (l_0) between the DCF tip and the GRIN lens, which determines the beam expansion before entering the lens and, therefore, the distal focusing condition (Figure S2(A)).
For the centered case, the distal focusing condition is characterized by the beam width (w) and the working distance (z). As shown in Figure S2(B,C), increasing l_0 produces a monotonic decrease in z, whereas

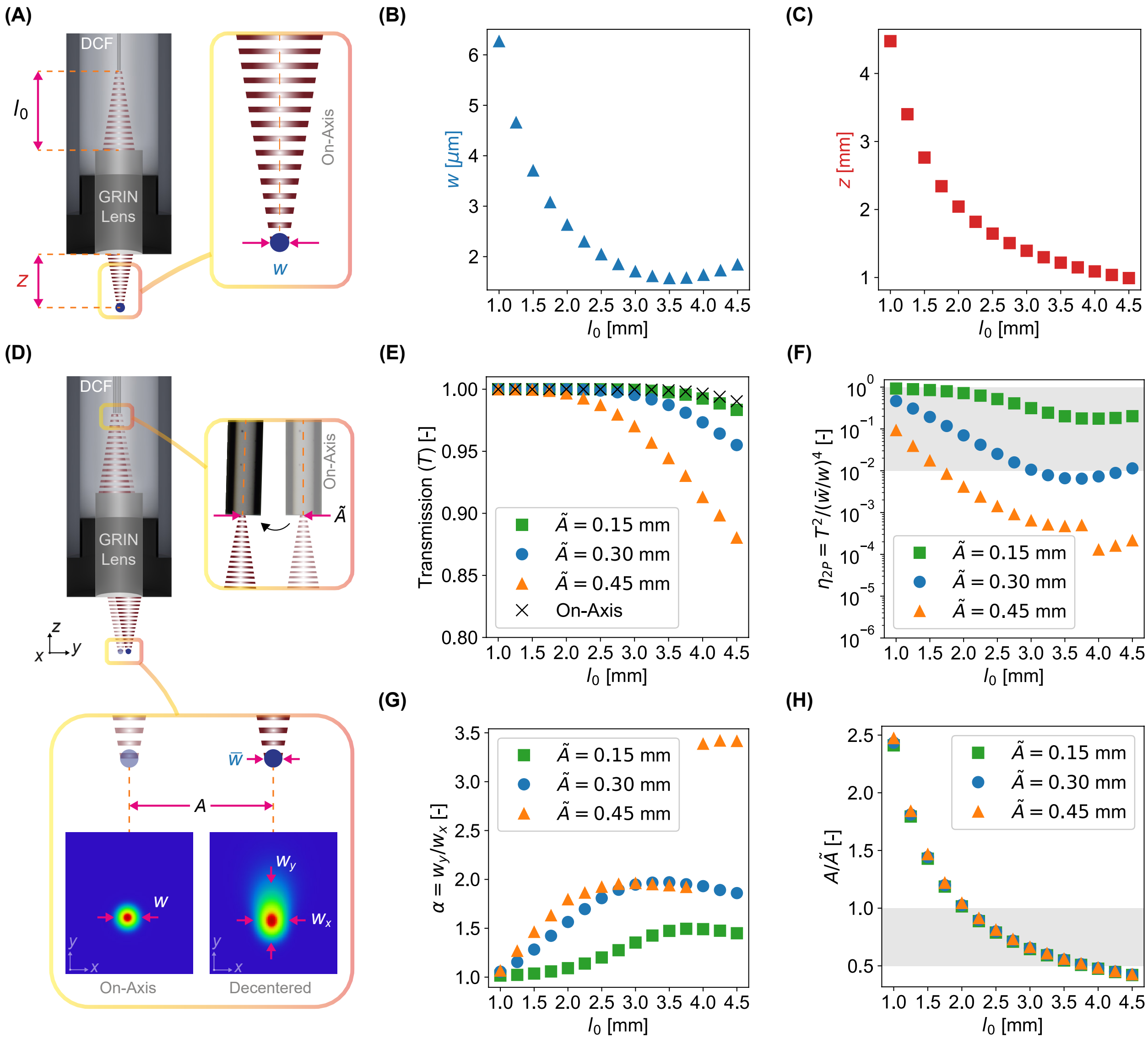

Figure S2: Optical beam propagation and off-axis beam behavior in the TPE probe. **(A)** Schematic of the centered optical configuration. The GRIN lens focuses light exiting the double-clad fiber (DCF). The axial fiber-to-lens offset (l_0) determines the focusing geometry, while the beam width and working distance characterizes the distal focusing condition. **(B)** Simulated centered beam width (w) as a function of l_0. **(C)** Working distance (z) versus l_0. **(D)** Schematic of the decentered case. A lateral input decenter ($\tilde{A}$) is used to emulate off-axis beam positions during DCF scanning, producing a distal optical displacement (A). The decentered spot is characterized by different widths along the x/y-axes (w_x and w_y) with average width ($\bar{w}$). **(E)** Normalized transmission (T) versus l_0 for different imposed decenters, including the centered case as reference. **(F)** Relative two-photon excitation proxy ($\eta_{2P} = T^2/(\bar{w}/w)^4$) versus l_0. The shaded region indicates the reference range $10^{-2} \leq \eta_{2P} \leq 1$. **(G)** Beam anisotropy ($\alpha = w_y/w_x$) versus l_0. **(H)** Scan transfer ratio ($A/\tilde{A}$) versus l_0. The shaded region indicates the reference range $0.5 \leq A/\tilde{A} \leq 1$.

w shows a local minimum within the analyzed range around $l_0 = 3.5$–4.0 mm. This indicates that increasing l_0 initially improves the centered focus, but beyond this range, the spot no longer becomes narrower. As later shown in Figure S2(E), this regime is associated with a progressive loss of on-axis transmission.
To evaluate off-axis performance, a lateral input decenter ($\tilde{A}$) is imposed at the DCF side, producing a distal optical displacement (A) and an off-axis focal spot with axis-dependent widths (w_x and w_y) (Figure S2(D)). The average off-axis width is defined as $\bar{w} = (w_x + w_y)/2$. Figure S2(E) shows that the normalized transmission (T) already decreases in the on-axis case as l_0 increases, and that this reduction becomes more pronounced for larger imposed decenters. This behavior is consistent with a progressively larger fraction of the beam approaching the GRIN boundary at larger l_0, which penalizes off-axis trans-

mission more strongly.
To summarize the off-axis implications for two-photon operation, a relative two-photon excitation proxy is defined as

$$\eta_{2P} = \frac{T^2}{(\bar{w}/w)^4}.$$

This quantity combines delivered power and focal broadening into a compact proxy for relative off-axis two-photon excitation efficiency. As shown in Figure S2(F), η_{2P} decreases with increasing l_0 and with larger imposed decenters, indicating that stronger off-axis penalties accompany a narrower on-axis spot. The shaded region in Figure S2(F) marks the selected reference range $10^{-2} \leq \eta_{2P} \leq 1$, corresponding to the retention of 1% to 100% of the on-axis excitation proxy. This range is used as a practical design reference to avoid configurations with severe off-axis degradation.
Beam anisotropy is quantified by $\alpha = w_y/w_x$, which captures off-axis spot distortion. Figure S2(G) shows that α increases with both l_0 and $\tilde{A}$, indicating progressively stronger elongation of the focal spot. The abrupt deviation observed for the largest imposed decenter ($\tilde{A}$ = 0.45 mm) near l_0 = 4 mm is consistent with strong clipping at the GRIN boundary, which also explains the discontinuity visible in η_{2P} in Figure S2(F).
The scan-transfer ratio ($A/\tilde{A}$) describes how proximal beam motion is converted into distal optical displacement and is therefore directly related to the accessible scanned field of view. As shown in Figure S2(H), $A/\tilde{A}$ decreases with increasing l_0, indicating reduced scan transfer at larger fiber-to-lens offsets. The shaded region in Figure S2(H) marks the selected reference range $0.5 \leq A/\tilde{A} \leq 1$, which is used as a design criterion to retain at least half of the imposed proximal scan amplitude at the distal field.
Taken together, these simulations show that the distal optical design is not selected by optimizing a single on-axis metric. Although the centered beam width w continues to decrease up to larger l_0, this is accompanied by progressively stronger off-axis penalties in transmission, η_{2P}, beam anisotropy, and scan-amplitude ratio. The selected range of approximately l_0 = 2.5–3.0 mm is therefore chosen as a design compromise, where the centered spot remains sufficiently narrow, and the off-axis metrics still satisfy the selected reference criteria for excitation efficiency and scan amplitude.

Note S2: Scanning Characterization

The oscillatory scanning behavior of the assembled probe, including the GRIN focusing optics, is experimentally characterized to determine the operating conditions used for imaging. Sinusoidal voltages are applied to the orthogonal electrode pairs of the PZT tube, producing lateral oscillations of the fiber tip along the x and y directions. The corresponding driving signals are

$$V_x(t) = V_{ppx} \sin(2\pi f_x t), \qquad V_y(t) = V_{ppy} \sin(2\pi f_y t),$$

where V_{ppx} and V_{ppy} denote the peak-to-peak driving voltages, and f_x and f_y are the driving frequencies. The scanning range is first measured as a function of driving frequency at a fixed peak-to-peak voltage of 25 V. As shown in Figure S3(C), the scanner responses along both lateral axes exhibit resonance peaks near 810 Hz. These results are consistent with the cantilever resonance predicted by the mechanical analysis in Figure S4 for free fiber lengths (l_f) around the assembled value of 11.5 mm. For imaging operation, frequencies within the 820–840 Hz interval are selected, where the response remains stable and approximately linear.
Within this frequency range, the scanning range increases approximately linearly with the applied peak-to-peak voltage along both axes (Figure S3(D)). The slopes of the voltage–displacement response are extracted for different operating frequencies to quantify the achievable scan extent (Figure S3(E)).
Lissajous scanning trajectories are generated by selecting pairs of driving frequencies (f_x, f_y) with slightly different values. The selection procedure is summarized in Figure S3(F): candidate frequency pairs are constrained by the scanner voltage limit (V_{pp} < 200 V) and chosen such that their greatest common divisor satisfies $\gcd(f_x, f_y) = f_0$. Here, f_0 = 5 Hz is used as the fundamental scan frequency, providing a practical compromise between image rate and spatial coverage. The resulting trajectories are mapped to

the image grid to evaluate pixel coverage and sampling behavior (Figure S3(G)). The filling factor and scanning anisotropy are computed for all valid frequency pairs within the selected operating range (Figure S3(H)).

Based on this analysis, the frequency pair $(f_x, f_y) = (825, 820)$ Hz is selected as it is a valid pair throughout all entire analyzed scanning range (125–250 µm). For the final operating condition, we use the corresponding to peak-to-peak driving voltages of $V_{pp,x} = 147.4$ V and $V_{pp,y} = 122.5$ V. This configuration leads to a Lissajous scan with 232 and 316 µm along the two scanning axes.

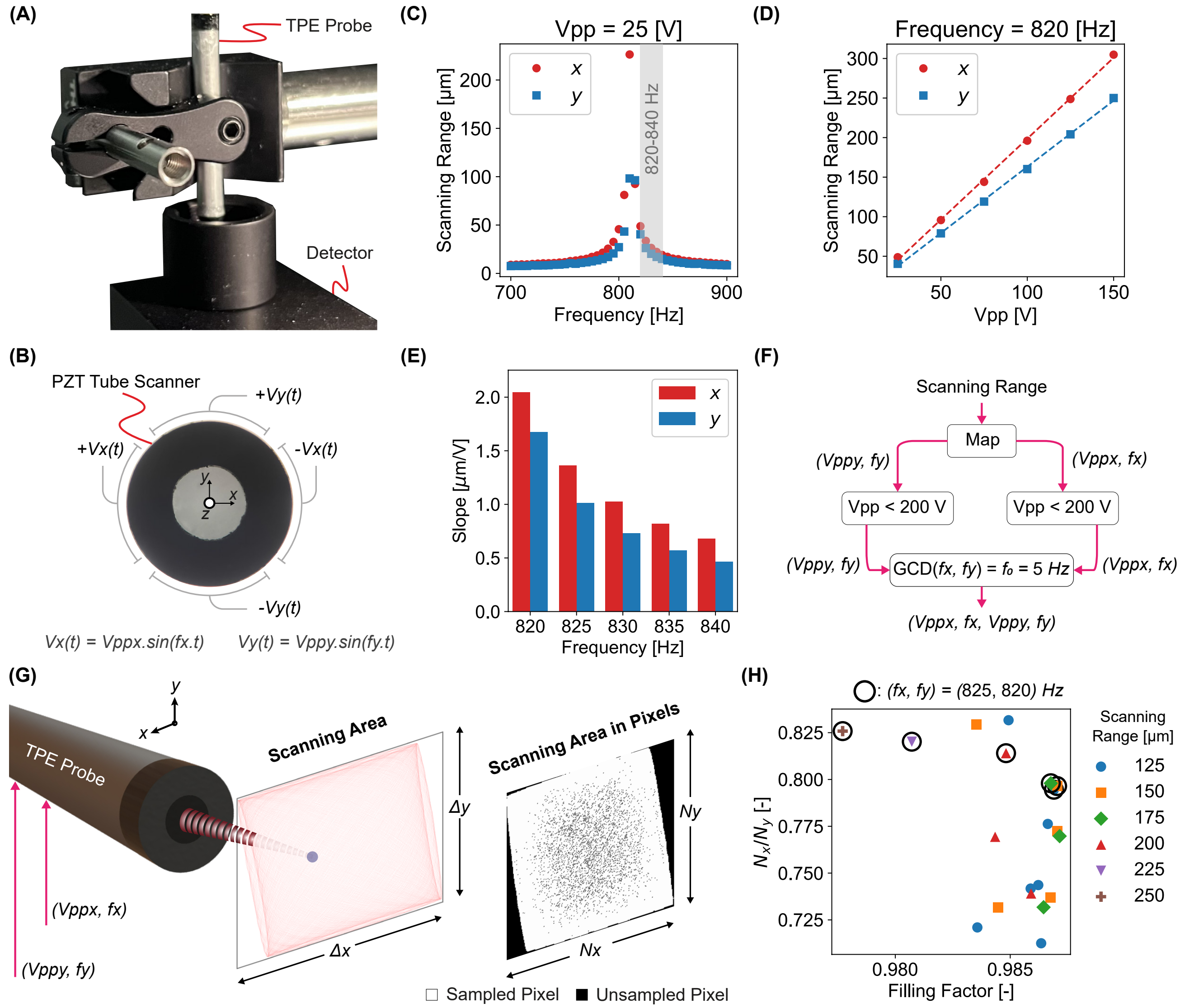

Figure S3: Oscillation and scanning characterization of the two-photon endomicroscopy (TPE) probe. **(A)** Experimental setup used to measure the fiber oscillations and scanning trajectory. **(B)** Cross-section of the PZT tube scanner showing the electrode configuration and sinusoidal driving voltages $V_x(t)$ and $V_y(t)$. **(C)** Scanning range as a function of driving frequency at $V_{pp} = 25$ V. The scanner exhibits a resonance near ∼810 Hz; the 820–840 Hz interval is selected for further analysis due to its approximately linear response. **(D)** Scanning range versus peak-to-peak voltage V_{pp} at $f = 820$ Hz for both x and y axes. **(E)** Slopes of the scanning range–voltage relationship within the 820–840 Hz interval. **(F)** Workflow for selecting pairs of driving frequencies (f_x, f_y) that achieve the desired scanning range while maintaining $V_{pp} < 200$ V. The greatest common divisor of (f_x, f_y) defines the Lissajous scanning frequency $f_0 = 5$ Hz. **(G)** Mapping of the Lissajous scanning trajectory to the pixel grid. N_x is fixed, while N_y is determined by the ratio $\Delta x/\Delta y$. **(H)** Scanning anisotropy versus filling factor for valid frequency pairs at scanning ranges between 125 and 250 μm with N_x=300 pixels. The pair $(f_x, f_y) = (825, 820)$ Hz (encircled) remains valid across the entire range.

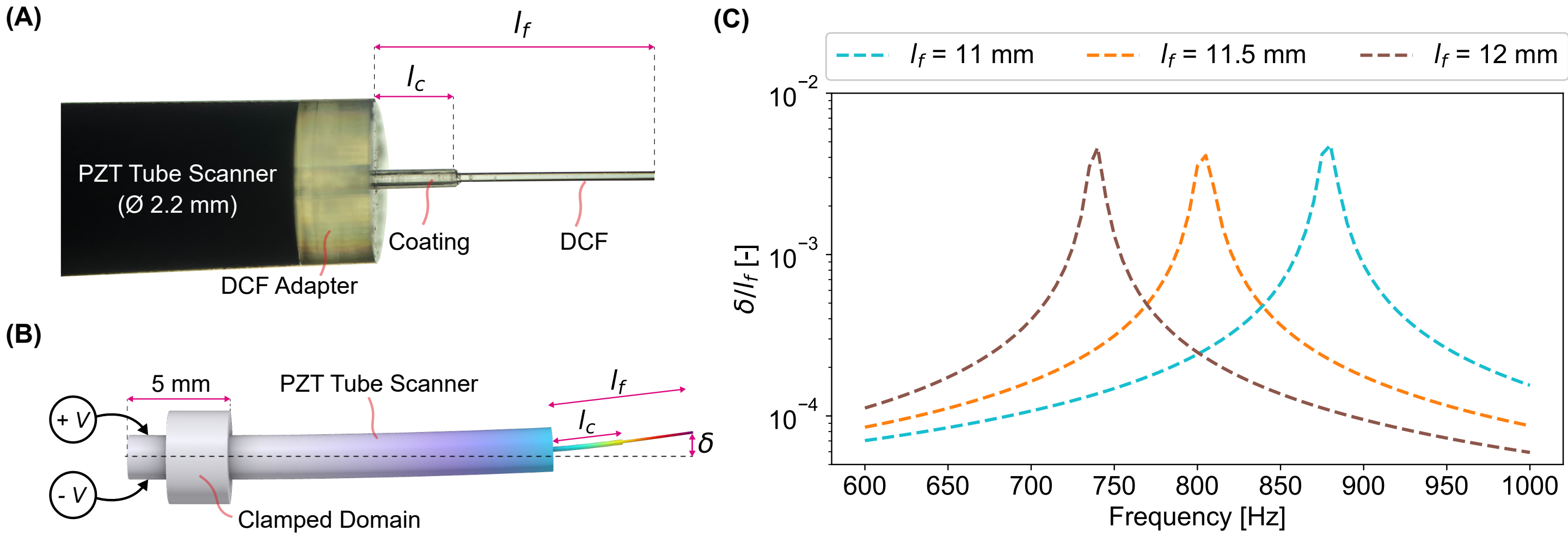

Figure S4: Resonant response of the PZT tube scanner as a function of free fiber length. **(A)** Photograph of the probe assembly showing the PZT tube scanner (outer diameter 2.2 mm), DCF adapter, coated fiber section, and double-clad fiber (DCF). The coating length (l_c) and free fiber length (l_f) are indicated. **(B)** Schematic representation of the PZT tube scanner operating as a cantilever. The clamped domain spans 5 mm, while the free fiber section (l_f) extends beyond the tube. Differential voltage applied to the PZT electrodes produces a lateral tip deflection δ. The coating length is defined as $l_c = 0.1, l_f$. **(C)** Simulated frequency response of the normalized tip deflection (δ/l_f) for three representative free lengths (l_f = 11.0, 11.5, and 12.5 mm). Increasing l_f shifts the resonant response toward lower frequencies, consistent with cantilever behavior.

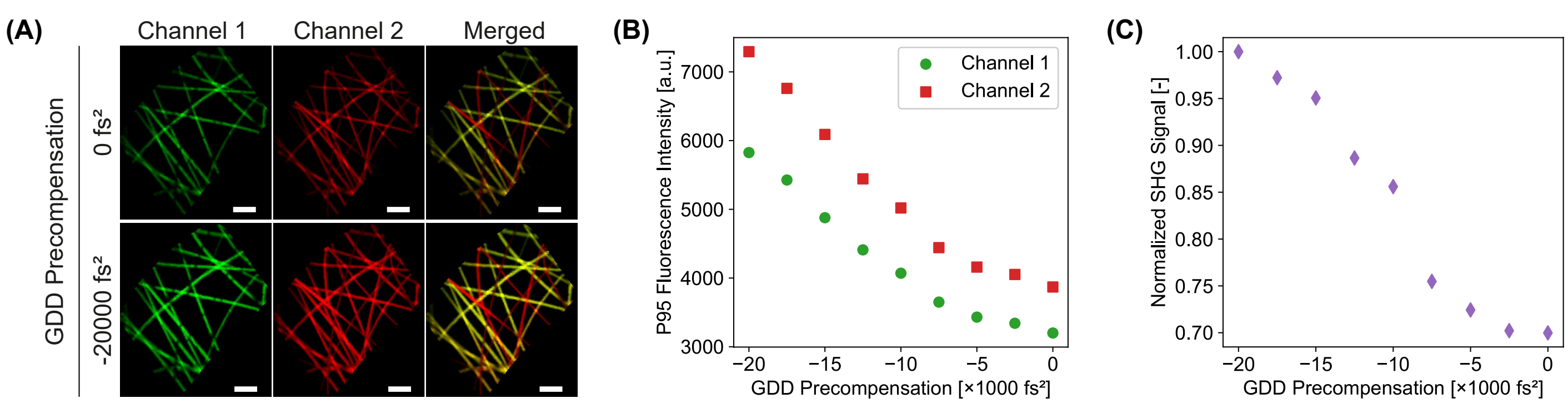

Figure S5: Effect of dispersion precompensation on nonlinear signal generation. **(A)** Two-color fluorescence images acquired without dispersion precompensation (0 fs^2) and with optimized precompensation (-20000 fs^2). Channel 1 (green) and Channel 2 (red) correspond to two spectrally separated fluorescence signals, and the merged images illustrate their spatial overlap. Improved signal brightness is observed when dispersion is compensated. Scale bars = 50 µm. **(B)** 95th percentile (P95) fluorescence intensity for both channels as a function of applied group delay dispersion (GDD) precompensation. Increasing negative GDD enhances the detected fluorescence signal, indicating improved pulse compression at the sample. **(C)** Normalized second-harmonic generation (SHG) signal as a function of GDD precompensation. The SHG response confirms that dispersion compensation increases peak pulse intensity, consistent with more efficient nonlinear excitation.

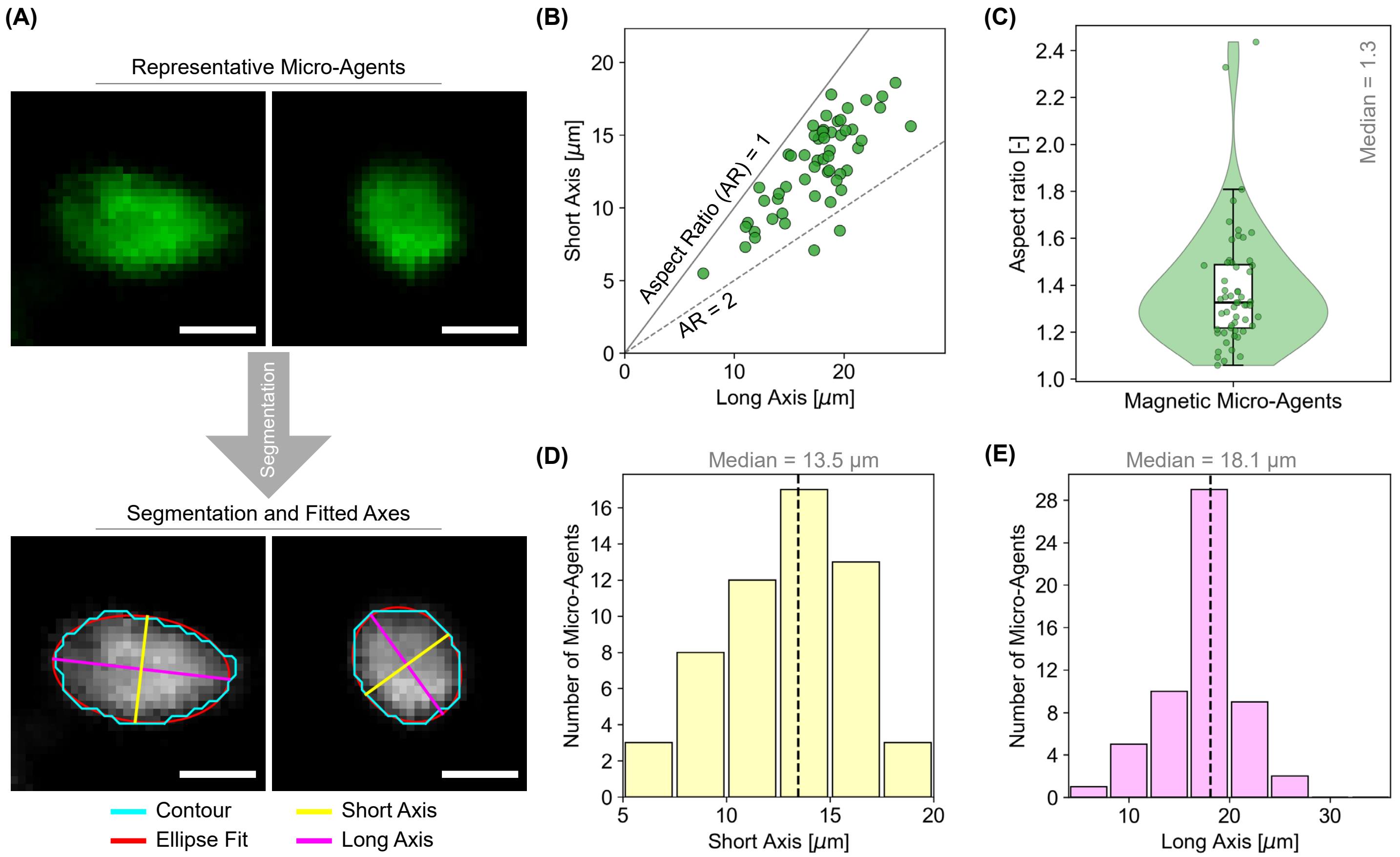

Figure S6: Size and morphology of magnetic micro-agents. **(A)** Representative two-photon images of magnetic micro-agents and the corresponding segmentation and ellipse fitting used to determine their dimensions. Cyan, red, magenta, and yellow indicate the detected contour, fitted ellipse, long axis, and short axis, respectively. Scale bars: 10 μm. **(B)** Short-axis dimension plotted against the long-axis dimension for individual micro-agents. The solid and dashed diagonal lines indicate aspect ratios of 1 and 2, respectively. **(C)** Distribution of the measured aspect ratios, showing individual observations and an embedded box plot. **(D,E)** Distributions of the short- and long-axis dimensions, respectively. Dashed vertical lines indicate the corresponding medians. Measurements comprise 56 individual micro-agents segmented across 10 distinct frames.

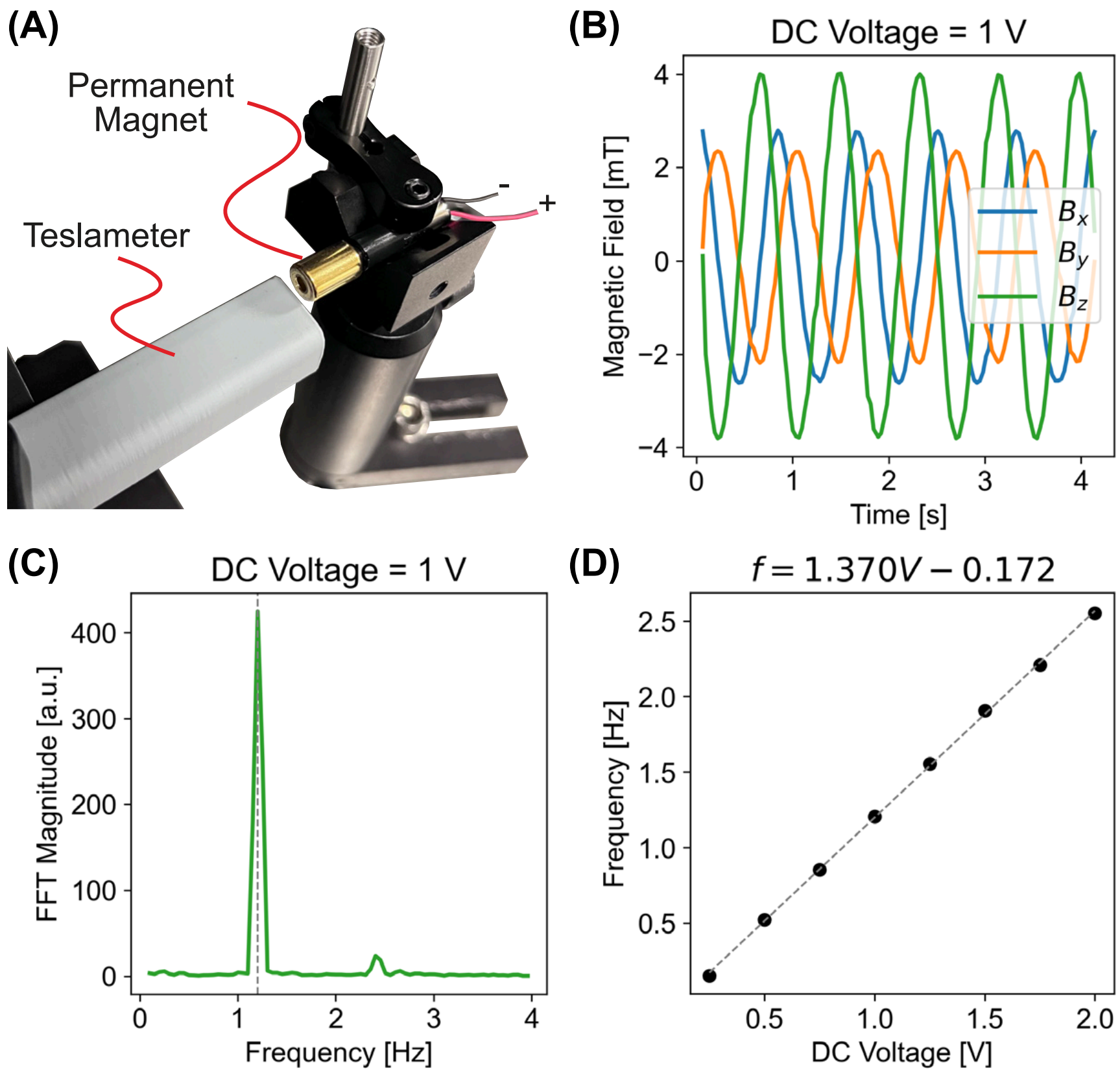

Figure S7: Magnetic field characterization and voltage-to-frequency mapping for micro-agent actuation. **(A)** Experimental setup used to measure the magnetic field generated by the rotating permanent magnet. A teslameter probe is positioned near the actuation region to record the time-varying magnetic field components. **(B)** Temporal evolution of the magnetic field components (B_x, B_y, B_z) measured at a DC voltage of 1 V applied to the motor. The oscillatory behavior arises from the rotation of the permanent magnet. **(C)** Fast Fourier transform (FFT) of the measured magnetic field signal, showing the dominant frequency component associated with magnet rotation. **(D)** Rotation frequency as a function of the applied DC voltage. The dashed line indicates a linear fit (f = 1.370V - 0.172), showing linear control of the actuation frequency through the motor driving voltage.

Note S3: Magnetic Field Gradient Estimate

The field gradient at the workspace was estimated from the measured rotating field and the manufacturer specifications of the ring magnet (remanence $B_r = 1.24$ T; outer diameter $D_\mathrm{o} = 6.8$ mm, inner diameter $D_\mathrm{i} = 3.0$ mm, height $h = 10$ mm). Approximating the magnet as a point dipole, its moment is

$$m = \frac{B_r V}{\mu_0}, \qquad V = \frac{\pi}{4}\left(D_\mathrm{o}^2 - D_\mathrm{i}^2\right) h \approx 0.29\ \mathrm{A\,m^2}. \tag{1}$$

The field of the dipole at position $\mathbf{r}$ is

$$\mathbf{B}(\mathbf{r}) = \frac{\mu_0}{4\pi r^3}\left[3(\mathbf{m}\cdot\hat{\mathbf{r}})\,\hat{\mathbf{r}} - \mathbf{m}\right]. \tag{2}$$

As the diametrically magnetized magnet rotates, the field at the workspace traces a planar ellipse whose minor semi-axis amplitude A_min fixes the magnet–workspace distance,

$$A_\mathrm{min} = \frac{\mu_0 m}{4\pi r^3} \quad \Longrightarrow \quad r = \left(\frac{\mu_0 m}{4\pi A_\mathrm{min}}\right)^{1/3} \approx 9\ \mathrm{mm}. \tag{3}$$

Because the dipole field scales as r^{-3}, the corresponding gradient scales as r^{-4}, giving a representative magnitude

$$|\nabla \mathbf{B}| \sim \frac{3\,|\mathbf{B}|}{r} \sim 20\ \mathrm{T\,m^{-1}}, \tag{4}$$

which, because the field rotates, varies over each actuation cycle. As the recovered distance is comparable to the magnet dimensions, the point-dipole model provides an order-of-magnitude estimate rather than an exact value. In this rotating-field regime, micro-agent motion is governed primarily by magnetic torque ($\mathbf{m} \times \mathbf{B}$). To compare the magnetic torque scale with the gradient-force contribution over the micro-agent size, a characteristic length is defined as the arithmetic mean of the median short and long micro-agent axis dimensions ($L \approx 16\ \mu$m) (Figure S6(D)–(E)). Using this characteristic length, the ratio between the torque scale and the gradient-force contribution over the micro-agent size is

$$\frac{|\mathbf{B}|}{L\,|\nabla \mathbf{B}|} \approx 10^2, \tag{5}$$

indicating that torque-driven reorientation, not gradient pulling, dominates the actuation.

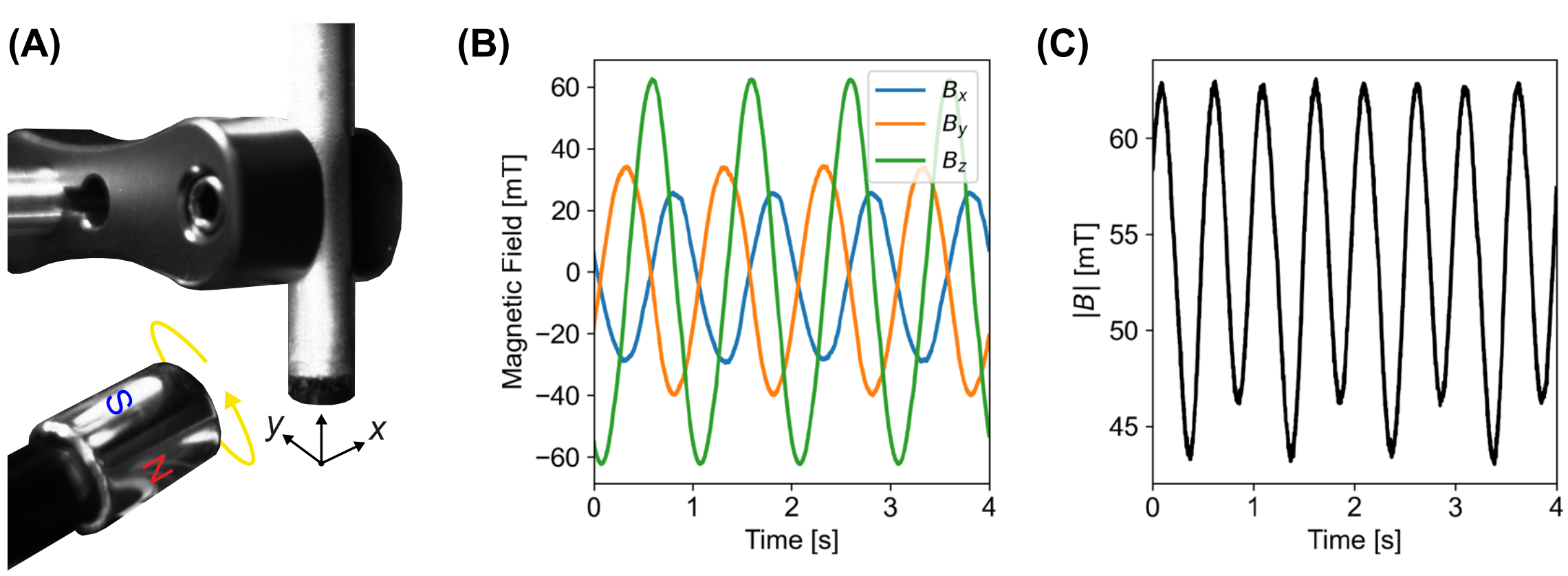

Figure S8: Rotating magnetic field generation for micro-agent actuation. **(A)** Experimental configuration showing the rotating permanent magnet used to generate a time-varying magnetic field at the workspace. The rotation axis and coordinate system are indicated as yellow and black arrows, respectively. **(B)** Measured temporal evolution of the magnetic field components (B_x, B_y, B_z) during magnet rotation, illustrating the periodic variation of the vector field. **(C)** Corresponding magnitude of the magnetic field ($|B|$) as a function of time, demonstrating the oscillatory field strength experienced at the measurement location.

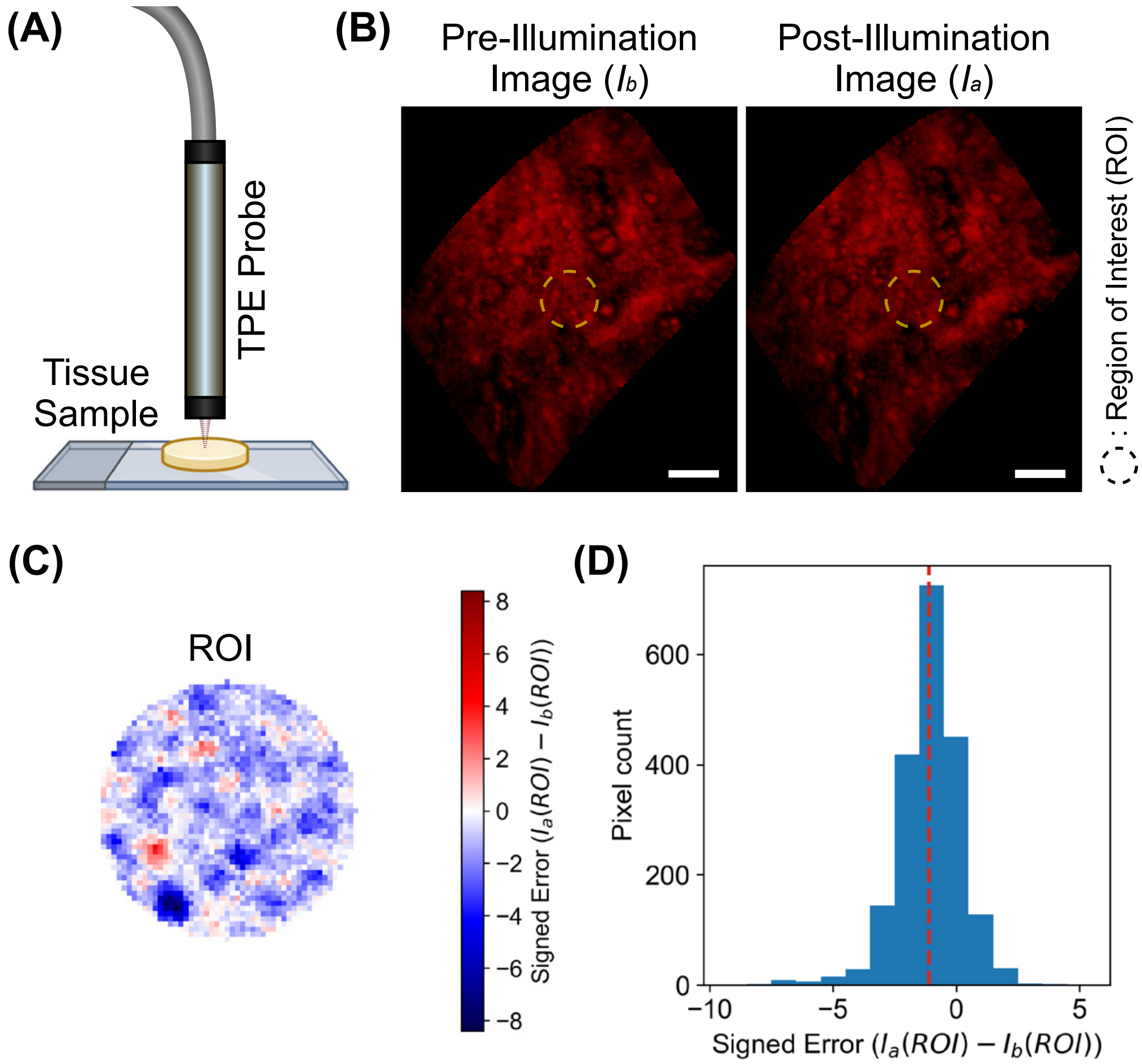

Figure S9: Evaluation of illumination-induced effects during stationary beam exposure. **(A)** Schematic of the experimental configuration used to assess potential illumination-induced changes. The TPE probe is positioned above the surface of a Rhodamine B–stained mammary gland tissue sample, and the excitation beam (240 mW average power, 77 MHz repetition rate) is parked at the tissue surface for 3 min, exceeding the highest imaging power used in this study (200 mW). **(B)** Representative pre-illumination image (I_b) and post-illumination image (I_a) acquired before and after stationary beam exposure. A circular region of interest (ROI, 50 px diameter) centered at the beam location is used for the quantitative analysis. For each condition, 10 frames are recorded and averaged to reduce frame-to-frame fluctuations. Scale bars = 50 µm. **(C)** Mean signed error map within the ROI, computed as I_a(ROI) - I_b(ROI). **(D)** Histogram of pixel-wise signed intensity differences within the ROI. The dashed red line indicates the mean of the distribution (-1.11 on a 0–255 scale), corresponding to a 9.5% decrease in mean intensity relative to the pre-illumination value

Table S1: Representative performance of external and local imaging modalities relevant to micro-agents.

**Modality**	**Distal diameter**	**Spatial resolution**	**Useful depth**	**FOV**	**Temporal resolution**	**Contrast**	**Label-free**	**Depth sectioning**[e]	**Ref.**
**External deep-tissue imaging**									
Clinical B-mode ultrasound	–	∼0.1–1 mm	Several cm to > 10 cm	cm-scale	Tens of fps	Acoustic backscatter	Yes	Echo delay	[1, 2] [3, 4]
Photoacoustic computed tomography	–	125–255 μm in-plane	cm-scale (up to 4 cm)	∼3.9–4.8 cm in-plane	10–50 Hz (2D)	Optical absorption (spectral)[d]	Yes	Acoustic arrival time	[5, 6] [7, 8]
Magnetic particle imaging (MPI)	–	∼1–5 mm	No tissue attenuation[a]	cm-scale	Tens of volumes s^{-1}	Magnetic tracer	No	Magnetic spatial encoding	[9, 10]
**Local endoscopic/endomicroscopic imaging**									
Photoacoustic endoscopy	∼0.8–3.2 mm[f]	Lat.: ∼10–300 μm; axial: ∼30–60 μm	∼1–3 mm (tissue-dependent)	mm-scale (360° scan)	∼2–50 fps	Optical absorption (spectral)[d]	Yes	Acoustinc time-of-flight	[11, 12]
Wide-field fluorescence endoscopy	Platform-dependent	Working-distance dependent	Surface	mm–cm	Video-rate	Fluorescence (multicolor)	Possible[b]	None	[13, 14] [15]
Confocal endomicroscopy	∼0.9–2.7 mm	Lat.: ∼0.7–3.5 μm; axial: ∼3–15 μm	∼40–250 μm	∼240–700 μm	∼8–12 fps	Fluorescence / reflectance	Possible[b]	Confocal pinhole rejection	[16, 17] [15]
Endoscopic optical coherence tomography	∼0.9–3.2 mm	Lat.: ∼20–40 μm; axial: ∼5–15 μm	∼1–2 mm	mm-scale (360° scan)	∼40–200 fps	Interferometric backscatter	Yes	Coherence gating	[18, 19] [20]
**Two-photon endomicroscopy (TPE)**	∼2.4–5.8 mm	Lat.: ∼0.6–3.1 μm; axial: ∼3–10 μm	∼100–200 μm; 300 μm[c]	∼80–750 μm	∼0.7–26 fps	Nonlinear fluorescence (multicolor)	Possible[b]	Nonlinear focal excitation	[21, 22, 23] [24, 25, 26] [27, 28, 29] [30, 31, 32] [33]

[a]MPI signal is not attenuated by intervening tissue, although spatial resolution, sensitivity, and accessible volume remain dependent on tracer properties and scanner geometry.

[b]"Possible" denotes that endogenous fluorescence or reflectance can generate contrast without an administered agent. Target-specific molecular contrast generally requires an exogenous agent.

[c]The 300 μm TPE result was demonstrated using enhanced fluorescence collection. Compact fiber-scanning systems in scattering tissue more commonly operate over approximately 100–200 μm. The reported 400 μm focus-tuning range is an addressable axial range rather than a measured tissue-penetration depth and is therefore not listed as useful depth.

[d]Spectral optoacoustic discrimination requires sufficiently distinct absorption spectra and multispectral acquisition; it is not directly equivalent to fluorophore-channel separation.

[e]Depth sectioning denotes the mechanism used to assign signal to depth or restrict contributions from out-of-focus regions. Confocal imaging filters out-of-focus fluorescence at detection, whereas TPE limits its generation through nonlinear focal excitation.

[f]The photoacoustic-endoscopy diameter range refers to compact catheter or working-channel probes. Larger capsule- and MEMS-based prototypes are not included in this table.

References

[1] J. Powers, F. Kremkau, *Interface Focus* **2011**, *1*, 4 477.

[2] A. Carovac, F. Smajlovic, D. Junuzovic, *Acta Informatica Medica* **2011**, *19*, 3 168.

[3] F. Šuligoj, C. M. Heunis, S. Mohanty, S. Misra, *IEEE Transactions on Biomedical Engineering* **2022**, *69*, 12 3739.

[4] S. Pane, M. Zhang, V. Iacovacci, L. Zhang, A. Menciassi, *APL Bioengineering* **2022**, *6*, 3.

[5] L. Li, L. Zhu, C. Ma, L. Lin, J. Yao, L. Wang, K. Maslov, R. Zhang, W. Chen, J. Shi, et al., *Nature biomedical engineering* **2017**, *1*, 5 0071.

[6] L. Lin, P. Hu, J. Shi, C. M. Appleton, K. Maslov, L. Li, R. Zhang, L. V. Wang, *Nature communications* **2018**, *9*, 1 2352.

[7] L. Li, L. V. Wang, *BME Frontiers* **2021**, *2021* 9823268.

[8] W. Choi, D. Oh, C. Kim, *Journal of Applied Physics* **2020**, *127*, 23.

[9] S. Harvell-Smith, L. D. Tung, N. T. K. Thanh, *Nanoscale* **2022**, *14*, 10 3658.

[10] A. C. Bakenecker, A. von Gladiss, H. Schwenke, A. Behrends, T. Friedrich, K. Lüdtke-Buzug, A. Neumann, J. Barkhausen, F. Wegner, T. M. Buzug, *Scientific Reports* **2021**, *11*, 1 14082.

[11] H. Guo, Y. Li, W. Qi, L. Xi, *Journal of Biophotonics* **2020**, *13*, 12 e202000217.

[12] R. Ansari, E. Z. Zhang, A. E. Desjardins, P. C. Beard, *Light: Science & Applications* **2018**, *7*, 1 75.

[13] M. Goetz, T. D. Wang, *Gastroenterology* **2010**, *138*, 3 828.

[14] J. A. Stibbe, P. Hoogland, F. B. Achterberg, D. R. Holman, R. S. Sojwal, J. Burggraaf, A. L. Vahrmeijer, W. B. Nagengast, S. Rogalla, *Molecular Imaging and Biology* **2023**, *25*, 1 18.

[15] Y. Tang, S. Anandasabapathy, R. Richards-Kortum, *Molecular Oncology* **2021**, *15*, 10 2580.

[16] J. M. Jabbour, M. A. Saldua, J. N. Bixler, K. C. Maitland, *Annals of biomedical engineering* **2012**, *40*, 2 378.

[17] Z. He, P. Wang, Y. Liang, Z. Fu, X. Ye, *Journal of Healthcare Engineering* **2021**, *2021*, 1 7594513.

[18] M. J. Gora, M. J. Suter, G. J. Tearney, X. Li, *Biomedical optics express* **2017**, *8*, 5 2405.

[19] O. C. Mora, P. Zanne, L. Zorn, F. Nageotte, N. Zulina, S. Gravelyn, P. Montgomery, M. De Mathelin, B. Dallemagne, M. J. Gora, *Biomedical Optics Express* **2020**, *11*, 3 1231.

[20] D. Li, D. Dong, W. Lam, L. Xing, T. Wei, D. Sun, *IEEE Transactions on Biomedical Engineering* **2019**, *67*, 8 2349.

[21] Y. Wu, Y. Leng, J. Xi, X. Li, *Optics express* **2009**, *17*, 10 7907.

[22] K. Murari, Y. Zhang, S. Li, Y. Chen, M.-J. Li, X. Li, *Optics letters* **2011**, *36*, 7 1299.

[23] D. Y. Kim, K. Hwang, J. Ahn, Y.-H. Seo, J.-B. Kim, S. Lee, J.-H. Yoon, E. Kong, Y. Jeong, S. Jon, et al., *Scientific Reports* **2019**, *9*, 1 3560.

[24] V. Kučikas, M. P. Werner, T. Schmitz-Rode, F. Louradour, M. A. van Zandvoort, *Molecular Imaging and Biology* **2021**, 1–15.

[25] W. Liang, H.-C. Park, K. Li, A. Li, D. Chen, H. Guan, Y. Yue, Y.-T. A. Gau, D. E. Bergles, M.-J. Li, et al., *IEEE Transactions on Medical Imaging* **2020**, *39*, 12 3779.

[26] H.-C. Park, H. Guan, A. Li, Y. Yue, M.-J. Li, H. Lu, X. Li, *Optics Letters* **2020**, *45*, 13 3605.

[27] C. Wang, H. Liu, J. Ma, H. Cui, Y. Li, D. Wu, Y. Hu, D. Wu, Q. Fu, L. Liang, et al., *Optics Express* **2021**, *29*, 26 43124.

[28] A. Li, H. Guan, H.-C. Park, Y. Yue, D. Chen, W. Liang, M.-J. Li, H. Lu, X. Li, *Optica* **2021**, *8*, 6 870.

[29] W. Liang, D. Chen, H. Guan, H.-C. Park, K. Li, A. Li, M.-J. Li, I. Gannot, X. Li, *ACS Photonics* **2022**, *9*, 12 4017.

[30] H. Guan, W. Liang, A. Li, Y.-T. A. Gau, D. Chen, M.-J. Li, D. E. Bergles, X. Li, *Optics Letters* **2021**, *46*, 5 1093.

[31] C. Wang, H. Liu, J. Ma, Q. Fu, Y. Li, Y. Chen, Y. Gao, J. Tian, X. Luo, F. Yu, C. Zhao, R. Wu, A. Wang, L. Feng, *Photonics* **2023**, *10*, 3 342.

[32] B. Camli, L. Andrus, A. Roy, B. Mishra, C. Xu, I. Georgakoudi, T. Tkaczyk, A. Ben-Yakar, *Biomedical Optics Express* **2024**, *15*, 5 3163.

[33] Y. Liu, J. L. Huang, X. Li, *Optics Letters* **2025**, *50*, 13 4194.
